# An Effective Protocol for Proteome Analysis of Medaka (*Oryzias latipes*) after Acute Exposure to Ionizing Radiation

**DOI:** 10.3390/mps2030066

**Published:** 2019-07-30

**Authors:** Yeni Pérez-Gélvez, Shem Unger, Gerardo Gutiérrez-Sánchez, Robert Bridger, Olin E. Rhodes, Carl Bergmann

**Affiliations:** 1 Complex Carbohydrate Research Center, The University of Georgia, Athens, GA 30602, USA; 2 Department of Biology, Wingate University, Wingate, NC 28174, USA; 3 Exact Sciences Laboratories, 145 E Badger Rd Suite 100, Madison, WI 53713, USA; 4 Savannah River Ecology Laboratory, The University of Georgia, Aiken, SC 29802, USA

**Keywords:** in-gel digestion, ionizing radiation, medaka, *Oryzias latipes*, proteome

## Abstract

All terrestrial organisms are subject to evolutionary pressures associated with natural sources of ionizing radiation (IR). The legacy of human-induced IR associated with energy, weapons production, medicine, and research has changed the distribution and magnitude of these evolutionary pressures. To date, no study has systematically examined the effects of environmentally relevant doses of radiation exposure across an organismal proteome. This void in knowledge has been due, in part, to technological deficiencies that have hampered quantifiable environmentally relevant IR doses and sensitive detection of proteomic responses. Here, we describe a protocol that addresses both needs, combining quantifiable IR delivery with a reliable method to yield proteomic comparisons of control and irradiated Medaka fish. Exposures were conducted at the Savannah River Ecology Laboratory (SREL, in Aiken, SC), where fish were subsequently dissected into three tissue sets (carcasses, organs and intestines) and frozen until analysis. Tissue proteins were extracted, resolved by Sodium Dodecyl Sulfate-Polyacrylamide Gel Electrophoresis (SDS-PAGE), and each sample lane was divided into ten equal portions. Following in-gel tryptic digestion, peptides released from each gel portion were identified and quantified by Liquid Chromatography-Mass Spectrometry (LC-MS/MS) to obtain the most complete, comparative study to date of proteomic responses to environmentally relevant doses of IR. This method provides a simple approach for use in ongoing epidemiologic studies of chronic exposure to environmentally relevant levels of IR and should also serve well in physiological, developmental, and toxicological studies.

## 1. Introduction

Ionizing radiation (IR), from other than natural sources, has become an aspect of daily life over the course of the last century. While sites such as Fukushima and Chernobyl are well-known and well documented sources of exposure to radiation, there remain over 1000 locations within the United States alone that are contaminated with radiation and have yet to be sufficiently studied to fully understand the risk to human health and to the environment. Testing and manufacturing related to nuclear proliferation (for both energy and weapons) and rapid increases in the use of nuclear medicine [1], are becoming increasingly identified as sources of radionuclide contamination. Such contamination can have long lasting effects on public health and the environment, particularly in aquatic systems.

The effects of radionuclides on organisms can vary depending on the dose and exposure time and may result in changes in morphology and functional activity, both at the cellular and system levels. It is well documented, especially at high doses, that IR has detrimental effects on aquatic organisms. These include double-strand breaks (DSBs) and oxidative damage to DNA, genomic instability, alterations in RNA, proteins, and other metabolites, as well as bystander and transgenerational effects [2,3]. At low to intermediate doses of IR, proximal DSBs can lead to complex DNA damage, and have received heightened attention recently due to their correlation with cytotoxicity, increased risk of cancer, and mutagenesis [4,5,6]. Additionally, nontargeted effects (NTE), such as the bystander radiation response, low-dose hyper-radiosensitivity, and radiation-induced adaptive response may be present after low-dose exposure [7], confounding the interpretation of organismal responses to radiation [8]. Several studies in fish have demonstrated bystander effects in which a signal passed from a fish exposed to radiation to an unexposed fish induces responses in the recipient [9,10]. A recent study presented potential transgenerational bystander effects in fish and amphibian cells [11].

Organismal responses to IR include alterations in the expression and/or post-translational modifications of specific proteins in cells, tissues, and organic fluids such as serum, plasma, and urine [12,13,14,15,16]. However, it is unclear how these processes translate into the metabolic adaptations which underlie evolutionary change within species. Comparative proteomic studies strongly indicate that protein expression profiling is a vital tool for investigating responses of proteins of metabolic and structural importance subsequent to IR exposure. Proteomic profiling in model species exposed to elevated levels of IR, including mice, indicates numbers of significantly deregulated proteins. These proteins often are associated with metabolic processes, inflammatory responses, cytoskeletal structure, as well as various transcription factors [15,17]. Preliminary research using Medaka has detected proteomic changes following exposure to moderate to high levels of radiation, including significant alterations in the levels of proteins associated with DNA repair and cellular apoptosis [18].

The use of Medaka in genetics, biomedical, environmental, and ecotoxicological research has a long history [19,20,21,22,23]. Medaka is an ideal vertebrate species for proteomic studies due to its readily available genome sequence databases (~800 Mb), [24,25] which are vital to the successful interpretation of proteomic data. Protein expression profiling can identify significant changes in protein expression (biomarkers) associated with IR exposure level [26]. Transcriptomic analysis can provide similar information, but is blind to post-transcriptional and post-translational modifications of protein expression, which often produce metabolic adaptations of evolutionary consequence [27]. Therefore, proteomic analysis of Medaka to reveal responses and adaptations to environmentally relevant levels of IR not only contributes to our understanding of the potential health risks of low level IR exposure, but also serves to elucidate past evolutionary events and the future evolutionary potential of organisms.

The challenge, and the goal of this research, is to design an effective protocol for detecting relevant changes in proteins expressed in Medaka and other organisms exposed to IR. This paper presents such a protocol and provides another tool for analysis of acute or chronic exposure to environmental stressors.

## 2. Experimental Design

The methods described for this study provide a simple approach to detect proteomic responses to irradiation across different tissues (carcasses, organs and intestines) in Medaka. The in-gel digestion protocol described is an economical, easy, and reliable protocol that could be applied to other epidemiological studies with large sets of samples. We used the in-gel digestion method to compare proteins in control samples as well as samples irradiated at a moderate level (500 mGy), since previous research has shown that exposure to this level is high enough to induce detectable changes but low enough not to immediately kill fish [9,28,29]. Our goal was to ascertain the optimal protocol for assessing proteomic changes to environmentally relevant levels of IR by conducting an experiment with both sham control and 0.5 Gy of exposure. This comparative dataset provides a baseline for use in future physiological, developmental, and toxicological studies at levels of resolution that have previously been unattainable. This method uses state-of-the-art techniques to allow us to obtain robust results which we describe in detail as follows: (1) How exposure to moderate levels of IR was accomplished; (2) how to prepare samples for comparative analysis, including in-gel digestion; and (3) how the data were handled to obtain basic biological information. This protocol was developed for use in exploratory analysis after exposure to stressors such as IR. The results of this initial exploratory analysis also demonstrate the need for additional strategies to obtain a more detailed understanding of the organismal response.

### 2.1. Materials

- 12 wild-type adult Medaka (~6–8 months old) with a body weight of 0.45 + 0.01 g and a 14 h:10 h light-dark cycle at 25 °C;
- 4 liter of filtered water;
- Anesthetic solution: 250 mg/L of tricaine methane sulfonate (MS-222) (Fisher, Fair Lawn, NJ, USA; cat: NC0342409), buffered with sodium bicarbonate at or near to neutral pH;
- Liquid nitrogen;
- Methanol HPLC grade (Sigma-Aldrich, St. Louis, MO, USA; Cat. no.: 34860-4L-R);
- Homogenizing solution kept at 4 °C: Mix chloroform (Fisher, Fair Lawn, NJ, USA; Cat. no.: C606SK-4), methanol, and Milli-Q water at ratio of 2:4:1.5, respectively;
- Acetone (Fisher, Fair Lawn, NJ, USA; Cat. no.: C606SK-4);
- Tris-HCl buffer: 1 mM Tris base (Fisher Scientific, Fair Lawn, NJ, USA; Cat. no.: BP152-500), pH 7.2 and 2% SDS (Sigma-Aldrich, St. Louis, MO, USA; Cat. no.: L4390);
- Pierce BCA protein assay kit (Thermo Scientific Pierce, Rockford, IL, USA; Cat. no.: 23227);
- Bolt 4–12% Bis-Tris Plus gels (Thermo Scientific Pierce, Rockford, IL, USA; Cat. no.: NW04120BOX);
- Protein ladder (Thermo Scientific Pierce, Rockford, IL, USA; Cat. no.: LC5925);
- 2× Laemmli Sample Buffer (Bio-Rad, Hercules, CA, USA; Cat. no.: 1610737);
- Running buffer 1×: 50 mL of Bolt MOPS SDS running buffer (Thermo Scientific Pierce, Rockford, IL, USA; Cat. no.: B0001) mixed with 950 mL of deionized H_2_O (dH2O);
- Plastic container 12 × 8 × 3 cm, rinsed previously with 50% aqueous methanol twice and one time with 100% methanol;
- Instant Blue Coomassie (Expedeon, Cambridge, UK; Cat. no: ISB1L);
- Destaining solution: 50 mL methanol, 5 mL acetic acid (J.T Baker, Center Valley, PA, USA; Cat. no.: 9508-33) and 50 mL dH2O;
- Isopropanol (Fisher, Fair Lawn, NJ, USA; Cat. no.: BP2635-4);
- Ambic solution: 0.158 g of ammonium bicarbonate (Sigma-Aldrich, St. Louis, MO, USA; Cat. no.: AG141-500G) dissolved in 20 mL of dH2O to obtain a 100 mM solution;
- Acetonitrile HPLC grade (ACN) (Fisher, Fair Lawn, NJ, USA; Cat. no.: A988-4);
- 10 mM dithiothreitol (DTT): 0.015 g of DTT (Research Products International, Mount Prospect, IL, USA; Cat. no.: D11000-25) dissolved in 10 mL of Ambic solution;
- 55 mM iodoacetamide (IDA): 0.10 g of iodoacetamide (Sigma-Aldrich, St. Louis, MO, USA; Cat. no.: I1149-5G) dissolved in 10 mL of Ambic solution;
- Trypsin solution: 20 µg of trypsin (Promega, Madison, WI, USA; Cat. no.: V5111) in 1 mL of cold Ambic solution. Final concentration 20 ng/µL;
- Extraction solution: 50% ACN / 50% dH2O and 0.1% formic acid (Sigma-Aldrich, St. Louis, MO, USA; Cat. no.: F0507-500mL);
- Buffer A (0.1% aqueous formic acid);
- Buffer B (80% ACN, 0.1% aqueous formic acid).

### 2.2. Equipment

- 1.5 L rectangular plastic containers (Pentair, Apopka, FL, USA; Cat. no.: PCt10);
- 1300 Ci Cs-137 source irradiator from the calibration facility at the Savannah River Nuclear Solutions (SRNS) (Aiken, SC, USA);
- Pyrex petri dish 100 × 10 mm (Sigma-Aldrich, St. Louis, MO, USA; Cat. no.: CLS3160100);
- Stainless steel disposable scalpels (Integra Miltex, York, PA, USA; Cat. no.: 4-411);
- Dressing forceps (Integra Miltex, York, PA, USA; Cat. no.: 18-184);
- Dumont #3c forceps (Fine Science Tools, Foster city, CA, USA; Cat. no.: 11231-20;
- Clear zip bag, 3″W × 4″H, 2 mL (Action health, Bensenville, IL, USA; Cat. no.: 85251-85002;
- Stereo Microscope (Olympus, Shinjuku, Tokyo, Japan; Cat. no.: (model): SZ51);
- Mortar and pestle (CoorsTek, Golden, CO, USA; Cat. No.: 60316 and 60317);
- 5 ¾″ disposable Pasteur borosilicate pipets (Fisher Scientific, Fair Lawn, NJ, USA; Cat. no.: 13-678-20B);
- Glass tube 16 × 125 mm (Corning Incorporated, Corning, NY, USA; Cat. no.: 99447-16) rinsed twice with 50% aqueous MeOH and once with 100% MeOH;
- Vertical Rocker Roto-Shake Genie (Scientific Industries, Bohemia, NY, USA; Cat. no.: SI-1100),
- Allegra 6 refrigerated centrifuge (Beckman Coulter life sciences, Brea, CA, USA; Cat. no.: 366816);
- Heto vacuum centrifuge or speed vac (Heto-Holten A/S, Allerod, Denmark; Cat. no.: 23905B VR-maxi St.a.-1);
- Vortex Genie 2 (Fisher, Fair Lawn, NJ, USA; Cat. no: 12-812);
- Lyophilizer (Labconco corporation, Kansas City, MO, USA; Cat. no.: 7960040);
- Microcentrifuge tube pestle (USA Scientific Inc., Ocala, FL, USA; Cat. no.: 1415-5390), rinsed twice with 50% MeOH and once with 100% MeOH;
- Mini Gel Tank (ThermoFisher Scientific, Rockford, IL, USA; Cat. no.: A25977);
- Horizontal rocker platform (Bellco Biotechnology, Vineland, NJ, USA; Cat. no.: 7740-10010);
- Ziploc bags quart freezer, 7″W × 7^11^/_16_″H (S. C. Johnson & Son, Inc. Racine, WI, USA);
- Glass and razor precleaned with Isopropanol 100% (see Note 2);
- New clean 1.7 mL Eppendorf tubes (MIDSCI, St. Louis, MO, USA; Cat. no.: AVSS1700);
- Heating module (Thermo Fisher Scientific, Rockford, IL, USA; Cat. no.: Pierce Reacti-Therm heating stirring module 18900);
- Incubator (Fisher, Pittsburgh, PA, USA; Cat. no.: 151030513);
- Nanosep 0.2 uM centrifugal filter units (PALL Life Sciences, NY, USA; Cat. no.: ODM02C34);
- Centrifuge (Beckman Coulter life sciences, Brea, CA, USA; Cat. no.: Microfuge 18);
- Glass crimp top vials (Thermo Fisher Scientific, Rockford, IL, USA; Cat. no: C4012-15), rinsed with MeOH;
- Snap caps for glass vials (VWR, Rador, PA, USA; Cat. no.: 14235-494);
- HPLC Ultimate 3000 RSLCnano (ThermoFisher Scientific, San Jose, CA, USA; Cat. no.: ULTIM3000RSLCNANO) with a 15 cm C18 analytical PepMap column (Thermo Fisher Scientific, San Jose, CA, USA; Cat. no.: 160321);
- Orbitrap Fusion Tribrid mass spectrometer (Thermo Fisher Scientific, San Jose, CA, USA; Cat. no.: IQLAAEGAAPFADBMBCX).

## 3. Procedures

### 3.1. Exposure to Radiation (Time for Completion: 48 h)

1. Divide the fish into 2 groups: 6 adult fish for the control group and 6 for the treatment group.
2. Place each group in small plastic containers with 0.5 L of filtered water.
3. Expose the treatment group to ionizing radiation (0.5 Gy) at the Savannah River site calibration facility using a 1300 Ci CS-137 source calibrated to a dose rate of 0.028 Gy/minute, for a total exposure of 17.9 min obtaining a total dose of 0.5 Gy. An additional sham control group (no exposure) is subjected to the same protocol to account for handling stress.
4. After exposure fish are returned to the laboratory and kept in tanks for 24 h.

### 3.2. Dissection (for 10 Fish 2–3 h)

**Figure 1:**
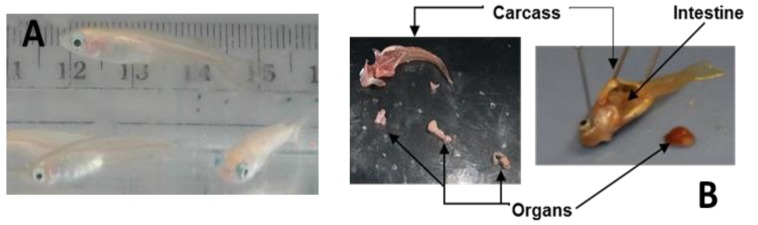
Medaka from the SREL facility. (**A**) Adult Medaka in a tank prior to the dissection and (**B**) dissected Medaka with arrows indicating carcass, organs, and intestines.

1. Euthanize the fish at 24 h post exposure according to the requirements of Animal Care and Use at the University of Georgia, AUP #A201305-018-Y1-A0 part C: Experimental procedures: “Euthanasia of animals: Animals sacrificed for proteomic tissue research (or sick diseased fish) will be euthanatized by an overdose via immersion in anesthetic solution. A concentration of 250–500 mg/L (5–10 times the anesthetic dosage) is effective for Medaka according to AVMA 2013 guidelines [30]. Medaka will be left in the anesthetic solution for a minimum of 10 min after cessation of opercular movement. Tissues used for the radiation/proteomics study will be frozen in liquid nitrogen and stored at −80 °C until extracted for proteomics analysis. Euthanasia of animals will occur only at the Savannah River Ecology Laboratory”.
  Note: The full AUP document can be found in the Supplementary Material File S1.
2. Place the fish into a glass petri dish (bottom or cover) and using a dissecting microscope open the fish with a scalpel, starting from the anus and continuing to the beginning of the head. Note: All the instruments and glassware must be clean, pre-washed with 50% methanol twice and 100% methanol once in order to avoid contamination of the samples. Use of plastic should be avoided, as it may result in contamination of the tissues with phthalates, complicating the mass spectrometry analysis.
3. Using dressing forceps, open the ventral area of the fish and take out the kidney, heart, liver, and gonads and put together in a plastic zip bag previously labeled. This will be the organs group. **CRITICAL STEP** The tissues have to be keep on ice until they are frozen to avoid degradation and/or expression of proteins associated with death.
4. Separate the intestines and stomach and place in another zip bag, and finally place the carcass (muscle, brain, eyes, gills, spinal cord, fins, and scales) in a third plastic bag. Figure 1 shows a dissected Medaka highlighting the different tissue groups.
5. Using liquid nitrogen freeze all the tissues for 30–60 s. **PAUSE STEP** The samples are stored at −80 °C until the next step.

### 3.3. Preparing Protein-Rich Powder (7 h)

The tissues need to be delipidated and prepared for total protein analyses as described previously [31] with some modifications. Note: Starting at this point all glassware must be new, and pre-washed twice with 50% methanol and once with 100% methanol to avoid contaminants that will interfere during the mass spectrometry analysis.

1. In a mortar and pestle, add the sample and 3 mL of the homogenizing solution using glass Pasteur pipets and homogenize the tissue.
2. Transfer the homogenized sample into a 15 mL glass tube. Rinse the mortar and pestle with 3 mL of the homogenizing solution and add the rinse to the homogenized sample.
3. Allow the sample to incubate at room temperature on a vertical rocker for 3 h.
4. Centrifuge the sample for 15 min at 4 °C at 3500 rpm. **CRITICAL STEP** The centrifugation generates heat, and thus refrigeration is necessary to avoid degradation of proteins.
5. Decant the supernatant (glycosphingolipids) and, then, dry down the protein pellet using vacuum centrifugation for approximately 15–20 min. **CRITICAL STEP** Do not over dry. Over drying will result in an incomplete/difficult homogenization and can cause degradation of the samples. Note: If there is any interest in analyzing the glycosphingolipids, the supernatant from step 5 and 8 should be preserved in a pre-cleaned glass tube, dried under nitrogen, and kept at −20 °C for further analyses.
6. Using Pasteur pipets, cover the sample in the bottom of the tube with homogenizing solution and incubate on the rocker for an additional 2 h at room temperature.
7. Centrifuge sample for 15 min at 4 °C at 3500 rpm.
8. Decant supernatant (glycosphingolipids) and, then, dry down the protein pellet using vacuum centrifugation.
9. Add 1 mL of cold (4 °C) Milli-Q water, and mix using the vortex. Add 4 mL of cold (4 °C) acetone, mix using the vortex, and incubate on ice for 15 min.
10. Centrifuge sample for 15 min at 4 °C at 3500 rpm. Decant supernatant into waste and dry down the protein pellet.
11. Repeat steps 9–10.
12. Freeze protein powder (−80 °C) and lyophilize overnight.
13. Once dry, store protein powder at −20 °C. **PAUSE STEP** the protein-rich powder can be keep at −20 °C for several months (In our case we have stored samples for up to 3 years with no significant change in the analyses). Note: the glass tubes have to be well capped to avoid humidity getting into the samples.

### 3.4. SDS-Electrophoresis (1.5 h)

1. Weigh 3–5 mg of protein-rich powder and resuspend with Tris-HCl buffer. Insoluble material is removed by centrifugation. Note: If necessary, use a microcentrifuge tube pestle before centrifugation to get a better homogenization of the sample. Prior to use, clean the pestle with 70% ethanol.
2. Determine the protein concentration using the Pierce BCA protein assay kit with bovine serum albumin as standard.
3. Prepare aliquots of 100 µg of protein and dry under vacuum centrifugation.
4. Add 15 µL of Milli-Q water to dissolve the dry sample and add the same volume of the 2× Laemmli Sample Buffer. Mix with the vortex and centrifuge. Note: The final volume cannot be more than 35 µL, this is due to the capacity of the loading wells being 40 µL. **CRITICAL STEP** Observe the color of the mix, if yellowish, add 2 µL 100 mM NaOH at a time and mix until it turns blue. Mix using the vortex and centrifuge again.
5. Boil the samples for 5 min and then put the samples into a refrigerator set at 7 °C for 5 min. Note: Be sure to cap the tubes well, or the sample will evaporate.
6. Add 10 µL of protein ladder in the first well. Add protein samples leaving an empty well between samples, this will simplify cutting out the individual gel sections for the in-gel digestion step.
7. Run the gel at 200 volts for 30–60 min.
8. Place the gel in a clean clear plastic container and add enough deionized water to cover it, swish back and forth 5 times. Pour out the water. Repeat the wash at least 3 times. Note: The plastic container must be dust and detergent free. It should be cleaned prior to use with 70% ethanol and allowed to dry.
9. Pour off the last water wash and add enough Instant Blue Coomasie stain to cover the gel, leave for 30 min to 1 h with gentle shaking. Note: Be sure that the gel can move freely in the staining solution to facilitate diffusion. Usually a 100 µg of protein will stain well after 30 min.
10. Discard the stain solution and wash 2–3 times with deionized water.
11. **PAUSE STEP** Keep the gel in water inside a Ziploc bag until the next step to avoid the gel drying out.

### 3.5. In-Gel Digestion (48 h after Full Distain of Gels Pieces)

1. Place the gel on sanitized glass. Use a razor blade to remove top and bottom of the gel. Note: prior to use, clean the glass with 50% methanol and 100% methanol, and then one time with isopropanol, then, let it dry.
2. Carefully cut each lane sample run into 10 equally sized sections and then cut each section into smaller pieces (1 × 1 mm^2^). Place all the gel pieces for each section into an Eppendorf tube. Note: Label the tube with sample and fraction information, i.e., control, fraction 5 can be CF5.
3. Add 500 µL destaining solution to the gels and put on a rocker. Replace the solution 2–3 times during the day or let it rock overnight at room temperature. Repeat this until the gels are completely destained. NOTE: The time to completely destain the pieces of gel will depend on the frequency of changing the destaining solution, but 24 h is the fastest that the gel pieces can be destained.
4. Once that the gel pieces are completely destained, remove destaining solution from each tube, using a different tip for each tube, then add 150 µL of HPLC grade water, and wait 5–10 min. Pull off water. Note: Starting at this point the tips and tubes used should be new and not been autoclaved, due to concerns of contamination that are detectable in the mass spectrometer.
5. Add 150 µL of 30% aqueous ACN and wait 5–10 min. Pull off the solution. Repeat.
6. Add 150 µL of Ambic solution, wait 5 min. Add 150 µL ACN 100% and wait 5–10 min. Pull off the solution.
7. Add 150 µL ACN, wait 5–10 min. Pull off the solution. Samples are then dried under vacuum centrifugation (50–60 min).
8. **PAUSE STEP** Properly capped tubes containing dried gel pieces can be stored at room temperature until the next step.
9. Add 150 µL of 100 mM DTT, incubate at 65 °C for 1 h. Remove the samples from bath, let cool to room temperature and pull off the solution.
10. Add 150 µL of 55 mM iodoacetamide for 1 h at room temperature in the dark. Pull off the solution.
11. Wash gel pieces with 150 µL of Ambic solution for 5–10 min. Add 150 µL ACN, wait 5–10 min. Pull off the solution.
12. Dry the gels under vacuum centrifugation for 45–60 min.

### 3.6. Tryptic Digestion (20 h)

1. Add trypsin solution 50:1 ww (protein/trypsin ratio) and, then, add enough Ambic to a final volume of 125 µL to ensure that the dry gel pieces are completely submerged. Note: For 100 µg of protein use 2 µg of trypsin (100 µL of trypsin solution). To ensure a better distribution and absorption of trypsin into the gels, we mix 100 µL of trypsin solution with 2.4 mL of Ambic to obtain a total of 2.5 mL (125 µL per sample × 20 samples = 2.5 mL). Vortex and add 125 µL of the mix.
2. Incubate over night at 37 °C (maximum 18 h).
3. After incubation, spin tubes, collect the supernatants and transfer each into a new prewashed tube (tube A). Change pipette tips between each tube.
4. Add 150 µL of extraction solution to the tubes containing the gel pieces and wait 5 min. Transfer the liquid to a fresh set of tubes (tube A). Repeat these extractions two more times.
5. Transfer all the liquid from the set of tube A’s to a set of Nanosep centrifugal filter units. Centrifuge at 12,000 rpm for 15–30 min.
6. The filtrates, containing tryptic peptides, are then dried on a speed vacuum, usually overnight. The samples can be stored at −20 °C until MS analyses.

### 3.7. LC-MS/MS Analysis of Tryptic Peptides (Mass Spectral Analysis) (8 h)

1. Suspend the dried peptide in 19 µL of buffer A and 1 µL of buffer B and, then, transfer the dilute peptides into glass crimp top vials pre-cleaned with methanol 50% and 100%.
2. Load the sample vials into the autosampler of an Ultimate 3000 LC System (Thermo Scientific Dionex).
3. Mass spectrometry parameters: Peptides are separated on a 15 cm C18 analytical PepMap Column (Thermo Fisher Scientific) and eluted into an Orbitrap Fusion Tribrid mass spectrometer (Thermo Fisher Scientific) utilizing a nanoelectrospray ionization source via a 90 min gradient of increasing buffer B at a flow rate of approximately 200 nL/min. The gradient goes from 1% to 99% of buffer B between 3–60 min and holds at 99% for 5 min, then, there is a ramp back down to 1% over 5 min and holding 1% for the last 20 min for equilibration. Full MS scans are acquired at 60K resolution and MS2 scans following collision-induced dissociation are collected in the ion trap for the most intense ions in top-speed mode within a three second cycle using Fusion instrumentation software (version 4.1, Thermo Fisher Scientific). Dynamic exclusion is utilized to exclude precursor ions from the selection process for 60 s following a second selection within a 10 s window. We perform ”blank-runs” (only buffer B) in between samples injections to ensure no carryover from sample to sample.
4. Results of the mass spectral analysis are in Raw format and are ready for the bioinformatics analysis that the user chooses. Below are summarized the bioinformatics and search options that we performed. Note: As an example, the raw data corresponding to the carcasses samples can be found in the public JPOST repository [32] under the Announced ID JPST000608.

### 3.8. Database Searching and Protein Identification (6 h)

1. Raw files obtained from the mass spectra analysis following each preparation/separation protocol were converted to mzXML files and then to pkl (peak list format) using Trans-Proteomic Pipeline Software (Seattle Proteome Center, Seattle, WA, USA). Each pkl file was searched for protein identification against concatenated database (normal and reverse database) containing proteins from the following species: *Oryzias latipes* and *Dario rerio*, from the Broad Institute and National Center for Biotechnology Information (NCBI) using MASCOT (Matrix Scientific, Boston, MA, USA). The reverse database is created by reversing all protein sequences from the target database using an in-house utility. Note: The concatenated fasta file can be found in the Supplementary Material File S2.
2. Mascot settings were as follows: tryptic enzymatic cleavages allowing for up to 2 missed cleavages, peptide tolerance of 20 parts-per-million, fragment ion tolerance of 0.5 Da, fixed modification due to carboxyamidomethylation of cysteine (+57 Da), and variable modifications of oxidation of methionine (+16 Da) and deamidation of asparagine or glutamine (+0.98 Da). Note: the pkl and mascot files corresponding to all the tissue groups can be found in the public JPOST repository under the Announced ID JPST000608.
3. Proteins were organized and filtered using a 1% protein false discovery rate applied, minimum 2 peptides, and 40 score in proteins via ProteoIQ software (Provalt_3.1.12_03-21-18, NuSep, Bogart, GA, USA) to obtain a nonredundant list of homologous protein groups [33], by loading Mascot target and decoy search files into the software program. (See Table 1 in results sections for an example of the list of some identified protein in Carcasses).

### 3.9. Protein Functional Annotation

Use the fasta sequence of each identified protein to obtain relevant biological information using the follow websites.

1. Gene Ontology terms are extracted from the Interpro and ProteoFun web sites (https://www.ebi.ac.uk/interpro/ and http://www.cbs.dtu.dk/services/ProtFun/).
2. Signal peptides in the deduced amino acid sequences are examined using the SignalP Web site (http://www.cbs.dtu.dk/services/SignalP/) and the SecretomeP 2.0 Web site (http://www.cbs.dtu.dk/services/SecretomeP/).
3. The family classification and functional category was obtained by using the pFam database (https://pfam.xfam.org/). (see Table 2 in results sections for an example of biological information of some proteins identified in carcasses).

## 4. Results and Discussion

**Figure 2:**
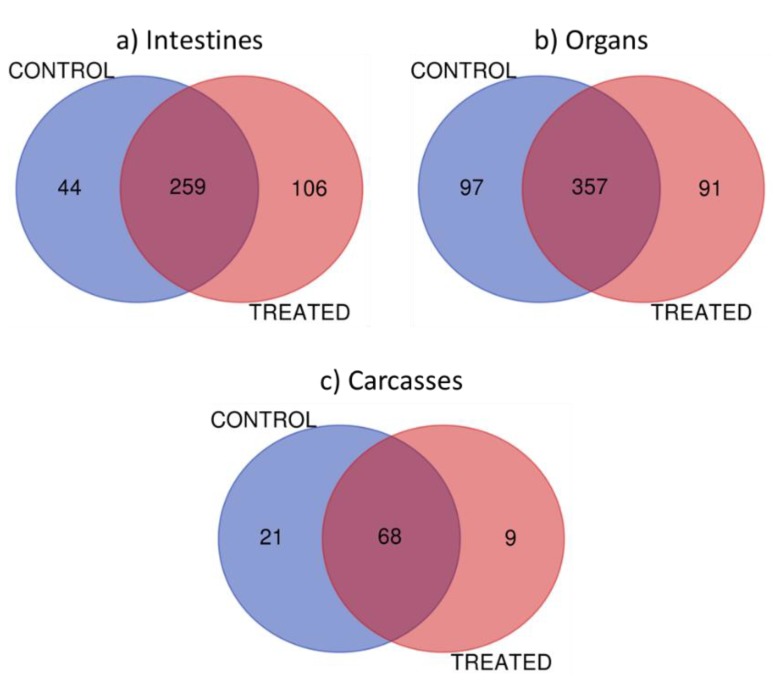
Venn diagram showing the number of identified proteins in Medaka. (**a**) Intestine with 409 total proteins identified, (**b**) organs with 545 identified proteins, and (**c**) carcasses with 98 identified proteins.

Three tissue sets were harvested (carcasses, organs, and intestines) from control and treatment fish. Proteomic search parameters were set to require a minimum of two peptides for each protein identification, in order to minimize false positives [34]. A total of 993 proteins in the control sample and 1004 in the treated samples were identified in the present study. Figure 2 presents the distribution of the number of proteins detected, showing the common proteins in the different tissues tested, as well as those which were unique from the irradiated or control samples. In total there were 409,545 and 98 proteins in intestines, organs, and carcasses, respectively, that fulfilled the search parameters. From these, there were 106, 91, and nine proteins in intestines, organs, and carcasses, respectively, which were identified as unique to the treatment group and might represent a response to radiation. Across all proteins, 33 were uncharacterized, which implies that they have been experimentally documented but are not characterized in biochemical terms [35]. Future investigation of these proteins (unique and uncharacterized) may open a door to a better understanding of the effects of IR and possibly to the bystander effects that occur after exposure; this is an area of study which is largely unexploited.

An example of the results from ProteoIQ is presented in Table 1 and the ProteoIQ information for all the proteins identified are available in the Supplementary Tables S1–S3 for carcasses, intestines, and organs respectively. Our results suggest that the protocol presented in this was able to identify changes at the protein level and the data obtained represent a valuable starting point for further research. From here, the data analyses will depend on the purpose of the study. For example, the spectral counts obtained after filtering the data with ProteoIQ can be used to evaluate the levels of protein expression and compare the control versus the treated samples. Relative spectral counts can be used to identify upregulation or repression in comparison to control using, for example, the relative spectral abundance factor (RSAF) [36,37]. In the current dataset, we observed that organs and intestines are more likely to be affected by IR exposure than carcasses, since only 39 proteins present in carcasses have an increase or decrease in relative spectral counts two-fold or greater, as compared to 200 in intestines and 264 in organs. The functional annotation described in procedures Section 3.8 usually is applied to upregulated, downregulated, or unique proteins to provide insight into those processes that may be impacted by the stressor (biological information of the proteins with greater than or equal to two-fold change is presented in Supplementary Tables S4–S6). An example of the result from the bioinformatics search of some upregulated proteins is presented in the Table 2.

**Figure 3:**
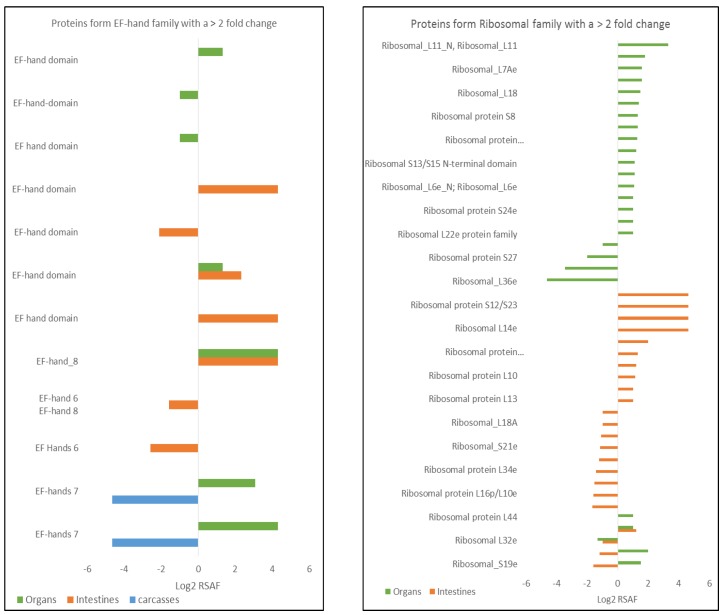
Histograms showing some proteins families with differential expression levels with ≥2-fold change. For graphic purposes proteins that were detected only in one set of samples were assigned a relative spectral abundance factor (RSAF) of 25, and as result of log2 transformation is equal to 4.64. Proteins that were identified only in the control sample are presented as −4.64 (–log_2_25.).

On the basis of the functional analysis (Supplementary Tables S4–S6), a few classes of proteins merit extra discussion due to their expression (up-/down-regulated) and/or high frequency of appearance in our results. Figure 3 shows two out of the four differentially expressed families discussed below. Sixteen proteins related to the EF-hand family exhibit a tissue-family dependent response to radiation. The EF-hand seven group showed repression in carcasses (two proteins only detected in control) but overexpression in organs (two proteins), while the EF-hand six group were repressed in intestines. Some proteins belonging to the EF-hand family can contribute to multiple processes like growth, cell motility, transcription, transduction, cell survival, and apoptosis [38], and are related with Alzheimer’s disease, Downs Syndrome, and ALS [39]. Proteins belonging to the ribosomal family were detected in organs (27) and intestines (26) with varying expression; in intestine these proteins are 50% repressed and 50% overexpressed, while in organs most of the ribosomal family proteins (21) are overexpressed. Ribosomal proteins can respond in different ways to IR exposure. Changes in the expression levels of proteins from this family as a result to exposure to IR have been reported [40,41], sometimes resulting in IR-sensitivity [42]. In addition, we detected proteins belonging to families that participate in dehydrogenase activity, such as Ldh, Aldedh, and ADH families. Proteins belonging to families with dehydrogenase function were repressed in treated organs but overexpressed in treated intestines. Previous studies have demonstrated that exposure to low and moderate levels of IR (0.02–1.0 Gy) reduces production of pyruvate dehydrogenase [14]. Reduction in enzymes like glucose 6-phosphate dehydrogenase increase the sensitivity to oxidative stress [43], which could increase sensitivity to IR. Our results suggest the same tendency toward repression of these families of proteins in treated organs. An increase in reactive oxygen species (ROS) in cells is a well know consequence after exposure to IR [44]. Lastly, 11 different proteins belonging to the Zona pellucida (ZP) family were overexpressed in our treated Medaka, mainly in the organs. The ZP domain is found in a variety of receptor-like eukaryotic glycoproteins that play fundamental roles in development, hearing, immunity, and cancer [45].

Our results demonstrate that our protocol identifies environmentally relevant IR-induced changes in the Medaka proteome. We detected members of several families of proteins that have been previously shown to respond to IR exposure, especially at high levels, providing us with additional confidence in our protocol’s effectiveness and indicating that environmentally relevant exposures may mimic high level exposure in some regards. The detection of differentially expressed proteins after exposure to environmentally relevant levels of IR provides us with a clearer understanding of organismal responses and adaptations to radiation. Our findings indicate certain protein families that may be critical to our understanding of the biological response of Medaka to environmentally relevant doses of IR, and they are likely candidates for future research in radiation biomarkers. Finally, the protocol presented here will enable studies of whole body response to IR and uncover trending expression changes during the course of chronic exposure to IR, ultimately leading to a more comprehensive understanding of the molecular and systemic impacts of IR.

## Figures and Tables

**Table 1:** Example of the outcome after the ProteoIQ filtering presenting the data for the top 30 proteins identified in carcasses.

Sequence Id	Sequence Name	Gene	Total Score	Total Peptides	Total Spectra	Control Score	Control Peptides	Control Spectral Count	Treated Score	Treated Peptides	Treated Spectral Count
gi|116062147|dbj|BAF34704.1|	fast skeletal myosin heavy chain isoform mMYH-7 [Oryzias latipes]	LOC110015468	7561.29	102	1935	7104.5	97	1061	5896.33	85	874
gi|116062139|dbj|BAF34700.1|	fast skeletal myosin heavy chain isoform mMYH-5 [Oryzias latipes]	LOC101163631	7442.38	100	1852	7069.86	96	1022	5726.27	82	830
gi|116062137|dbj|BAF34699.1|	fast skeletal myosin heavy chain isoform mMYH-6 [Oryzias latipes]	LOC111947749	7317.12	99	1755	6798.53	92	960	5818.96	84	795
gi|116062145|dbj|BAF34703.1|	fast skeletal myosin heavy chain isoform mMYH-3 [Oryzias latipes]	LOC101163661	6961.55	96	1640	6463.32	89	896	5464.79	80	744
gi|116062143|dbj|BAF34702.1|	fast skeletal myosin heavy chain isoform mMYH-2 [Oryzias latipes]	LOC101163414	6885.75	94	1697	6514.88	90	953	5332.89	78	744
gi|116062149|dbj|BAF34705.1|	fast skeletal myosin heavy chain mMYH-9 [Oryzias latipes]	LOC101163903	6816.79	92	1675	6449.61	88	933	5242.95	76	742
gi|1174695518|ref|XP_020560645.1|	myosin heavy chain, fast skeletal muscle-like [Oryzias latipes]	LOC101163903	5923.87	82	1426	5592.76	78	811	4426.38	66	615
gi|116062151|dbj|BAF34706.1|	fast skeletal myosin heavy chain isoform mMYH-11 [Oryzias latipes]	LOC101164155	5713.97	77	1363	5411.98	74	779	4382.73	64	584
gi|432868092|ref|XP_004071407.1|	myosin heavy chain, fast skeletal muscle [Oryzias latipes]	LOC101158198	5675.58	79	1400	5396.89	76	771	4490.4	65	629
gi|239735374|dbj|BAH70477.1|	myosin heavy chain embryonic type 1 [Oryzias latipes]	mmyhemb1	4594.2	64	1254	4369.27	62	703	3572.81	55	551
gi|1040677427|ref|XP_017208743.1|	myosin heavy chain, fast skeletal muscle [Danio rerio]	LOC113076616	4293.4	60	1003	4122.75	59	574	3208.54	49	429
gi|528483089|ref|XP_001339206.5|	myosin heavy chain, fast skeletal muscle [Danio rerio]	myhb	3651.99	51	839	3460.57	48	477	2656.29	42	362
gi|239735378|dbj|BAH70479.1|	myosin heavy chain larval type 2 [Oryzias latipes]	mmyhl2	3505.06	45	752	3270.63	44	422	2650.25	37	330
gi|239735376|dbj|BAH70478.1|	myosin heavy chain larval type 1 [Oryzias latipes]	mmyhl1	3434.68	44	767	3216.83	43	425	2614.22	37	342
gi|28422303|gb|AAH46881.1|	Zgc:66156 protein, partial [Danio rerio]	zgc:66156	3092.15	43	586	2917.85	42	340	2349.64	36	246
gi|432864495|ref|XP_004070322.1|	intermediate filament protein ON3-like [Oryzias latipes]	LOC101167707	1287.9	21	120	1007.83	16	59	1107.19	18	61
gi|432920251|ref|XP_004079911.1|	alpha-actinin-3 [Oryzias latipes]	actn3	1221.75	22	61	1108.52	20	44	286.58	6	17
gi|1174681026|ref|XP_020566641.1|	actin, alpha skeletal muscle isoform X1 [Oryzias latipes]	acta1	1102.66	17	268	833.62	12	148	917.13	14	120
gi|1174662893|ref|XP_020566626.1|	myosin-7 [Oryzias latipes]	LOC100125526	1055.2	16	265	979.22	15	148	793.85	13	117
gi|432895621|ref|XP_004076079.1|	creatine kinase M-type [Oryzias latipes]	LOC101166239	932.48	13	135	900.94	13	88	528.82	8	47
gi|1174683558|ref|XP_020557031.1|	vitellogenin 1 isoform X1 [Oryzias latipes]	ol-vit1	903.99	14	71	712.57	11	29	647.26	10	42
gi|1207193593|ref|XP_021329509.1|	actin, alpha cardiac muscle 1 [Danio rerio]	LOC108941121	862.86	13	213	760.34	11	118	668.01	10	95
gi|432852666|ref|XP_004067324.1|	tropomyosin alpha-1 chain isoform X1 [Oryzias latipes]	LOC101164789	855.51	14	127	825.11	14	75	658.6	11	52
gi|432922695|ref|XP_004080348.1|	sarcoplasmic/endoplasmic reticulum calcium ATPase 1 isoform X1 [Oryzias latipes]	LOC101171864	821.21	13	96	575.85	8	46	630.84	11	50
gi|190338754|gb|AAI63562.1|	Myosin, heavy polypeptide 6, cardiac muscle, alpha [Danio rerio]	myh6	806.87	13	183	742.39	12	99	633.76	10	84
gi|432864501|ref|XP_004070325.1|	keratin, type II cytoskeletal 8-like isoform X1 [Oryzias latipes]	LOC101168366	799.55	14	43	516.97	9	24	605.5	11	19
gi|432847946|ref|XP_004066228.1|	keratin, type I cytoskeletal 13-like [Oryzias latipes]	LOC101159648	795.99	12	91	729.77	12	38	644.12	9	53
gi|765137894|ref|XP_011480537.1|	creatine kinase M-type [Oryzias latipes]	LOC101163677	794.05	11	122	789.15	11	85	410.13	6	37
gi|1174691476|ref|XP_020559720.1|	tropomyosin alpha-1 chain [Oryzias latipes]	LOC112151854	697.97	12	102	687.97	12	62	466.42	8	40
gi|628601863|ref|NP_001278765.1|	fructose-bisphosphate aldolase A [Oryzias latipes]	aldoa	697.38	9	133	622.22	8	68	593.27	9	65

Score: Refers to either Mascot ion score, SEQUEST Xcorr, or tandem hyper score. Total protein score: Sums peptide score for all peptides matching to a protein.

**Table 2:** Biological information of some overexpressed proteins detected in Medaka organs after acute exposure to IR.

Sequence Id	Sequence Name	Gene	RATIO RSAF	pFAM	Secretome	Signal IP Score		Biological Process Involve in	Molecular Function Enables	Cellular Component Part of
gi|1174671686|ref|XP_004080935.3|	LOW QUALITY PROTEIN: Ras-related protein Rab-18 [Oryzias latipes]	rab18	NC	Ras family	0.377	0.118	NO	NP	3924 GTPase activity 5525 GTP binding	NP
gi|765158221|ref|XP_011488823.1|	uncharacterized protein LOC101162088 [Oryzias latipes]	LOC101162088	NC	Zona pellucida-like domain	0.535	0.735	YES	GO:2000344 positive regulation of acrosome reaction GO:0005803 egg coat formation GO:0007339 binding of sperm to zona pellucida	GO:0032190 acrosin binding	NP
gi|1174681297|ref|XP_020567346.1|	60S ribosomal protein L12 [Oryzias latipes]	rpl12	10	Ribosomal_L11_N, Ribosomal_L11	0.853	0.331	NO	GO:0006412 translation	GO:0003735 structural constituent of ribosome	GO:0005840 ribosome
gi|765151708|ref|XP_011486186.1|	parvalbumin beta-like [Oryzias latipes]	LOC101173896	8.5	EF-hands 7	0.328	0.176	NO	NP	GO:0005509 calcium ion binding GO:0046872 metal ion binding	GO:0005737 cytoplasm GO:0005634 nucleus
gi|157278241|ref|NP_001098220.1|	ZPC domain containing protein 4 precursor [Oryzias latipes]	LOC100049336	5.33333	zona pellucida	0.721	0.659	YES	GO:0007339 binding of sperm to zona pellucida GO:0035803 egg coat formation GO:2000344 positive regulation of acrosome reaction	GO:0035804 structural constituent of egg coat GO:0032190 acrosin binding	NP
gi|1174689816|ref|XP_011474004.2|	60S ribosomal protein L22-like [Oryzias latipes]	LOC101166956	3.5	Ribosomal_L22e	0.75	0.128	NO	GO:0033077 T cell differentiation in thymus GO:0006412 translation	GO:0003723 RNA binding GO:0003735 structural constituent of ribosome	GO:0005840 ribosome
gi|432867558|ref|XP_004071242.1|	cytochrome c oxidase subunit NDUFA4 [Oryzias latipes]	LOC101155111	3	B12D	0.884	0.239	NO	GO:0002290 electron transport chain GO:1902600 proton transmembrane transport	GO:0004129 cytochrome-c oxidase activity	GO:0016020 membrane GO:0016021 integral component of membrane GO:0005751 mitochondrial respiratory chain complex IV
gi|765145559|ref|XP_004077973.2|	60S ribosomal protein L18 [Oryzias latipes]	rpl18	2.8	Ribosomal_L18	0.614	0.202	NO	GO:0006412 translation	GO:0003735 structural constituent of ribosome	GO:0005840 ribosome
gi|1174693985|ref|XP_020560297.1|	myosin light polypeptide 6 isoform X1 [Oryzias latipes]	LOC101167617	2.5	EF-hand domain	0.4	0.106	NO	NP	GO:0005509 calcium ion binding	NP
gi|182889290|gb|AAI64893.1|	Calm1b protein [Danio rerio]	calm1	2.5	EF-hand domain	0.676	0.101	NO	GO:0019722 calcium-mediated signaling	GO:0005509 calcium ion binding GO:0046872 metal ion binding	NP
gi|1174655990|ref|XP_020564957.1|	uncharacterized protein LOC101172014 isoform X2 [Oryzias latipes]	LOC110014571	2.27273	NP	0.896	0.814	YES	NP	NP	NP
gi|765142574|ref|XP_011482440.1|	uncharacterized protein LOC101162625 isoform X2 [Oryzias latipes]	LOC101162625	2.26667	NP	0.687	0.738	YES	NP	NP	NP

NC: not detected in control NP: not predicted.

## Supplementary Materials

The following are available online at https://www.mdpi.com/2409-9279/2/3/66/s1, Table S1: Proteins identified in Carcasses in control and treated samples. Table S2: Proteins identified in Intestines in control and treated samples. Table S3: Proteins identified in Organs in control and treated samples. Table S4. Proteins with a ≥2 Fold change identified in Carcasses. Table S5 Proteins with a ≥2 Fold change identified in Intestines. Table S6. Proteins with a ≥2 Fold change identified in Carcasses. File S1: Animal Care and Use at the University of Georgia, AUP #A201305-018-Y1-A0. File S2: concatenated fasta file.
