# Supplementary material for: An Effective Protocol for Proteome Analysis of Medaka (Oryzias latipes) after Acute Exposure to Ionizing Radiation
Source: Methods Protoc. 2019 Jul 30;2(3):66. doi: 10.3390/mps2030066 (PMC6789492; doi:10.3390/mps2030066)
Supplement: Supplementary file 1 [file mps-02-00066-s001.zip › supplementary/A2013-05-018 Animal Care and Use at the University of Georgia..pdf]

Section 1: Overview

Animal Use Proposal

The Institutional Animal Care and Use Committee (IACUC) is responsible for ensuring that the use of animals at the University of Georgia is performed according to the highest standards and in an ethical manner. This responsibility is shared with university faculty, staff, and students. The use of animals at the University is a privilege, not a right.

Maintaining this privilege requires compliance with the following regulations, policies and guidelines:

- Animal Welfare Act Regulations
- Guide for the Care and Use of Agricultural Animals in Research and Teaching
- Public Health Service Policy on Humane Care and Use of Laboratory Animals
- The Guide for the Care and Use of Laboratory Animals
- U.S. Government Principles for the Utilization and Care of Vertebrate Animals Used in Testing, Research and Training.

This form is intended to facilitate review of requests to use animals for research and instruction. The review process has been designed to communicate rationale, justifications, methods, and materials for using animals through the Office of Animal Care and Use and Attending Veterinarians to the IACUC.

Artemis v1.1c 2013-11-20

Section 2: Key Information

**AUP Number:**	A2013 05-018-Y3-A1
**2.1 : Project Title:**	*A Proteomics approach to study the effects of low dose radiation on Medaka fish*
**2.2 : Principal Investigator:**	Olin Rhodes
**2.3 : Primary Funding Agency:**	*Department of Energy*

2.3.1 Aliases with Funding Agency

Protocol Title Alias: | Funding Agency: |

2.4 : Location
Will any aspect of the study (course) or animal husbandry be conducted at another institution?
Yes No

2.5 : Expiring Protocol
Will this AUP replace an expiring protocol? Yes No

2.6 : Purpose
What is the Purpose of this animal use protocol?
Instructional
Research

Section 3: Personnel and Qualifications

3.1: Personnel (US Government Principles, Principle #8 Guide, p 25-26 AW Regs 9 CFR Part 2, sec 2.31, sec 2.32)

Project Roster: Please provide the names of all the individuals who will work with animals on this project to the IACUC. Include yourself and any other investigators, student employees, post-doctoral fellows, staff research associates, post-graduate researchers, laboratory assistants, and others who will actually work with the animals. You do not need to include the staff of the facility in which your animals will be housed.

Occupation Health Program: Supervisors must enroll their employees in the OVPR Occupational Health and Safety Program. Please enroll personnel by having them complete a "Risk Assessment/Animal Contact Health Surveillance Questionnaire", available at the OHSP page.

Training: Supervisors are responsible for insuring that their employees are adequately trained both in the specifics of their job and in the requirements of the Federal Animal Welfare Act.

All individuals working with live vertebrate animals, including the protocol Principal Investigator (PI), must complete federally required training on the pertinent laws and regulations covered in the "IACUC 101" course and health and safety covered in "Staying Healthy While Working with Animals" and "Sharps."

The PI is responsible for keeping this roster for these individuals current. If staff is added or removed from this project, please modify the protocol to reflect this change; this is an administrative change and does not requires full IACUC review.

CURRENT ROSTER:
Olin Rhodes *(PI)* [-]

Describe this persons role, responsibilities and any relevant training.

Dr. Rhodes is the project PI and Director of the UGA Savannah River Ecology Laboratory. Dr. Rhodes is a Full Professor in the Odum School of Ecology and serves as the direct supervisor to Dr. Shem D Unger, who is a postdoctoral researcher at SREL. Dr. Rhodes is a population genetecist with 30 years of experience working with wildlife. He will be responsible for experimental design, overseeing research, supervising as a PI.

Training Courses Completed:

Course Name	Completion Date	CEUs
Continuing Education for Animal Research Credit	2019-03-11	1.00
UGA IACUC 101	2018-11-28	1.00
Sharps Training	2018-09-19	0.50
OHSP Decline to Participate	2016-06-08	0.00
Research Occupational Health Enrollment	2016-06-08	0.00
Continuing Education for Animal Research Credit	2016-03-01	1.00
UGA IACUC 101	2016-01-21	1.00
UGA IACUC 101 Refresher	2016-01-21	1.00
UGA IACUC 101	2013-02-13	1.00
Staying Healthy while Working with Laboratory Animals	2013-02-13	1.00

REBECA JUAREZ [-]

Can edit this submission form and draft amendments/renewals for this protocol: ○ Yes ◉ No

Describe this persons role, responsibilities and any relevant training.

Becky Juarez is a Research Technician in Dr. Rhodes laboratory with extensive experience (~1 year full time) in Medaka Culture. She will be responsible for day to day fish culture and maintenance of all fish habitats. Becky has a MS degree in wildlife ecology and management.

Training Courses Completed:

Course Name	Completion Date	CEUs
Occ Health Update	2016-11-30	0.00
Occupational Health and Safety Enrollment	2016-05-02	0.00
Staying Healthy while Working with Laboratory Animals	2016-04-21	1.00
UGA IACUC 101	2016-04-20	1.00

Xiaoyu Xu [-]

Can edit this submission form and draft amendments/renewals for this protocol: Yes No

Describe this persons role, responsibilities and any relevant training.

Dr. Xu will be responsible for designing and assisting with exposure experiments for medaka to mercury. Dr. Xu has extensive experience with analysis of mercury exposure in aquatic organisms and has designed and undertaken experiments involving aqueous exposure of medaka to mercury as a postdoc.

Training Courses Completed:

Course Name	Completion Date	CEUs
Research Occupational Health Enrollment	2019-04-18	0.00
UGA IACUC 101	2019-04-17	1.00
Sharps Training	2019-02-25	0.50
Staying Healthy while Working with Laboratory Animals	2019-02-25	1.00
Occupational Health and Safety Enrollment	2016-04-28	0.00
Staying Healthy while Working with Laboratory Animals	2016-04-17	1.00
UGA IACUC 101	2016-04-17	1.00

Section 4: Project Objective, Significance and Animal Use Justification

4.1: Objective (US Government Principles, Principle # 2 Guide, p 12, 25)
["Why are you doing this experiment with animals and what do you propose to learn?"]
Please provide a brief statement, limited to 300 words, outlining the objectives of the procedures in this protocol.

- This must include a statement of your **experimental hypothesis or teaching objectives.**
- This must be in LAY TERMINOLOGY, understandable by someone with a high school education, with no scientific jargon.
- Please define all abbreviations/acronyms the first time they are used and explain medical terms.
- Please do not submit your grant proposal abstract for this section.

The primary objective of this study is to discover what proteins are affected by chronic, low level irradiation exposure in Japanese Medaka. We will use a proteomics approach to identify protein expression using the Savannah River Ecology Laboratory's Par Pond Low Dose Irradiation Facility (LoDIF) and laboratory techniques at the Complex Carbohydrate Research Center. The SREL LoDIF is an outdoor mesocosm system equiped with radiation sources to study exposure in aquatic organisms. This unique facility is an array of 40 outdoor mesocosms equiped with cesium-137 irradiation sources and unexposed controls, which provide exposure levels of 0, 2, 20, and 200 mGy/d mean exposure. Each mesocosm holds 965 L of water in a flow through system and 10-12 medaka are maintained in plastic 5 gallon buckets beneath sources during operational experiments. Additional controls are maintained in Aquatic Habitats (AHAB) systems with the adjacent Par Pond Laboratory.

Medaka rearing, experimental exposure, and euthanasia will occur at SREL.

Medaka euthanasia will only occur at SREL. No euthanasia will occur at the CCRC. Only Tissue samples from deceased fish will be brought to the CCRC.

We hypothesize that protein expression will differ significantly between control and across levels of low irradiation. This project will ultimately lead to further research utilyzing modern proteomic/glycomic approaches for the study of bioligical mechanisms affected by low levels of irradiation.

4.2 : Significance (US Government Principles, Principle # 2 Guide, p 12, 25)
Please provide a brief statement of the relevance of contributions your work might make to human/animal well-being or the expansion of knowledge. This statement must provide a rationale for your proposed use of animals. This must be written in LAY TERMINOLOGY, understandable by someone with a high school education, with no acronyms or scientific jargon. Please do not submit your grant proposal abstract for this section. Define all abbreviations the first time they are used and explain medical terms. ["How will the proposed use of animals benefit human or animal health or expand knowledge?"]

This research project will utilize a model species, Medaka to assess the biological effects of low doses of exposure in the environment using Proteomics. This approach will enable us to determine which specific proteins are involved when aquatic organims are exposed to low doses of irradiation. Presently, there are very few studies on chronic, low levels of radiation using current proteomics approachs. Studies conducted under this AUP will contribute to our understanding of these biological processes.

4.3 : Justification for the Use of Animals (US Government Principles, Principle #3 Guide, p 12, 25 AW Regs 9 CFR Part 2, sec 2.31)
Please provide a brief statement justifying your use of animals in the proposed project: ["Why must you use animals in this work?"]
Note: The response needs to be an explanation on the use of a live animal instead of a non-animal alternative (not just a justification for the species).

Using animals is the only way to determine the effects on animals. Japanese Medaka have long been used as a model species for research due to their ability to be kept and maintained under a variety of laboratory conditions. Moreover, the Medaka Genome has been fully described, enabling our research in Proteomics to utilize this knowledge and investigate the effects of protein expression at relevant low levels of radiation exposure. We must use animals to conduct effective studies in a scientifically rigorous way using a replicated experimental design.

Section 5: Animal Information

5.1 : Animal Information (Species, strain, use category and other parameters to be used on this protocol).
Provide details for each species to be used. Indicate the number of each species for which you wish to be approved for the 3 year lifespan of the AUP. If you wish to increase these numbers after this AUP is approved, an amendment will need to be submitted.

Animal Use Categorization
All animal use must be classified according to the anticipated level of perceived pain/stress/distress experienced by the animal(s). Animals must be classified under the highest category involved at any point. Procedures involving more than momentary or slight pain or distress must be discussed with the Attending Veterinarian in the planning of the research project.

Category A: Includes the use of animals in experimental procedures that would be expected to produce little or no pain or distress.

Category B: Includes the use of animals in procedures that involve minor pain or distress of short duration, or in procedures where pain and distress are alleviated through the use of anesthetics, analgesics, and/or tranquilizers.
Animals are not expected to show prolonged (days) clinical symptoms, other than some mild discomfort, during or after Type B procedures. Type B studies place an explicit responsibility on investigators to explore alternatives to the procedures which may cause pain or distress.

Category C: Includes the use of animals in procedures that involve potential for significant but unavoidable pain or distress to the animals. Type C studies place an explicit responsibility on investigators to explore alternatives to the procedures which may cause pain or distress. Convened quorum of the IACUC will review all Category C, multiple major survival surgeries, and any other proposal as requested by any member(s).

IMPORTANT: The reasons for using these procedures must be explained in a statement by you, the Principal Investigator, justifying their use. Provide scientific justification if withholding appropriate sedation, analgesia, or anesthesia.

Category D: Includes the use of invertebrate animals, cell cultures, embryonated eggs, certain biologic products, tissues obtained post-mortem from vertebrate animals (obtained at necropsy, slaughterhouses, meat markets), or observation of non-captive wildlife species where there is no contact with animals.

Species::(US Government Principles, Principle #3 Guide, p 12 AW Regs 9 CFR Part 2, sec 2.31 Internal record keeping/reporting data)	Fish
Strain:	Medaka
Highest Use Category:	Category A
Sex:	Both
Quantity (Numerical Only):	6000
Housing Location:	SREL FIELD SITE
Weight Range:	*4000*
Age Range:	*0-3 years*
Preferred Vendor:	Choose a Vendor...
Is the use of this species covered by the USDA Animal Welfare Act?	Yes No

5.2 : Justification of Animal Numbers (US Government Principles, Principle #3 Guide, p 12 AW Regs 9 CFR Part 2, sec 2.31 Internal record keeping/reporting data)

Group sizes are expected to represent the minimum number of animals that are needed to achieve the scientific or instructional objectives. Please indicate all the methods used to determine these numbers.

- [x] Statistical tools, such as power analysis, were employed to determine appropriate group sizes to ensure statistically valid outcomes
- [x] Previous experience with this experimental paradigm indicates this is the minimum number of animals needed.
- [x] Consultation with a biostatistician.
- [ ] This is a pilot study used to determine feasibility before proceeding with larger, more tightly controlled experiments.
- [ ] This is an instructional activity and this is the minimum number of animals needed based on class size and optimal student to instructor ratios.
- [x] Other

(Explain below):

Species: Medaka will be maintained at the Par Pond facility under appropriate conditions for experimental trials at the Low dose irradiation facility (LoDIF). Medaka fish (Oryzias laticeps) will be housed indoors and maintained for outdoor experiments. Wild type Medaka will be purchased from either a distributor in Japan, Aquatic Ecosystems Inc., or provided by Dr. Kullman at North Carolina State University. Medaka have served as an experimental model since the early 1900's (Yamamoto 1975) and are one of the most widely used fish species for embryological, toxicological, and genomic studies. This species has numerous advantages for experimental laboratory and field studies, including tolerance for a wide variety of water conditions, small size (average length of 25 to 50 mm and weight ~0.7 to 0.8 grams), short generation time (~6 to 8 weeks), and prolific capacity to reproduce (6 to 30 eggs per day; 3,000 eggs per female/breeding cycle and males capable of fertilizing eggs from several females (Yamamoto 1975, Kinoshita et al. 2012).

*Amount of Medaka needed: We are requesting to maintain 2,000 fish annually over the next three years. Each experiment conducted at the LoDIF requires ~576 fish under full use (12 fish per bucket * 6 treatments (1 High, 1 Medium, 1 Low exposure, 2 outdoor controls, 1 indoor control) * 8 blocks). We expect to be performing multiple experiments over the next three years as well as maintaining a minimal number of adult and juvenile fish on hand for culturing purposes in multiple AHAB's. We will need to maintain laboratory cultures, multigenerational lines of experimental fish for ongoing research, as well as fish for subsequent experiments as dictated by research needs. Each experiment may consist of up to ~576 adults exposed for several weeks at the LoDIF. We may also perform additional experiments on ~576 juveniles, or ~576 eggs as determined by the results of our preliminary experimental approach. Because Medaka life span under laboratory conditions can vary from ~12 months to 22 months (Ding et al. 2010, Hawkins et al. 2013), we will be housing fish at various life stages (eggs, juveniles, adults) over the course of three years in order to replace aging fish, maintain breeding pairs, and have an adequate number of fish for experimental purposes. Therefore, we expect to perform 2 full experiments per year (~1,200 fish) and maintain a culture (~800 fish/year) as needed.*

53 : Justification for species selection (US Government Principles, Principle #3 Guide, p 12 AW Regs 9 CFR Part 2, sec 2.31 Internal record keeping/reporting data)

The species has been selected because (check all that apply)

- [x] Previous work in the biomedical literature validates the use of this species as an animal model for this disease or biological process.
- [x] This is the lowest sentient species that provides appropriate size, tissue or anatomy for the proposed work.
- [x] There is a large body of existing research that would need to be repeated if another species was used instead.
- [x] Availability of reagents or research tools necessary for this research is unique to this species.
- [ ] Characteristics of the species make it uniquely suited for the proposed research.
- [ ] Other

54 : Will a breeding colony be maintained? (Guide, p 25 AW Regs 9 CFR Part 2, sec 2.31)
Definition of a breeding colony: animals mated repeatedly to produce multiple groups of offspring for ongoing research projects and to maintain the colony. This does not include a single mating to produce embryos or one group of offspring for a specific research project.

(●) Yes ( ) No

5.4.1 (a):

If you are going to be breeding animals to be used in multiple protocols, including this one, or sharing animals with other investigators, please submit a separate breeding colony protocol.

For additional information regarding breeding please see: IACUC SOP #52: Breeding Rodents

Breeders:

No Strains Chosen

Offspring:

No Strains Chosen

5.4.1 (b):

Specify what parameters you will assess to ensure that the animals are healthy.

Check all that apply:

☑ Activity ☑ Appearance ☑ Appetite (Requires method to assess individual animal intake-- not usually selected for group-housed, ad libitum fed animals) ☑ Excreta ☑ Respiratory pattern ☑ Temperature ☑ Laboratory tests or other evaluation (describe below):

We will monitor water level, perform regular water changes, monitor nitrite, ph, ammonia, and temperature.

5.4.1 (c):

Describe how many animals you anticipate breeding over the next 3 years, including all parents and offspring. These numbers should correspond with the animal numbers requested above and as described in your experimental protocol: (US Government Principles, Principle #3 Guide, p 12 AW Regs 9 CFR Part 2, sec 2.31)

Up to 2,000 per year (6,000 total over 3 years) as needed for maintaining Medaka for experimental trials at the Savannah River Ecology Laboratory Par Pond facility.

5.4.1 (d):

Describe known or anticipated health or well-being issues with the animals you are proposing to breed or their offspring: (Guide p 28)

Adults and offspring will be monitored for health status as outlined in our procedures (8.2). Eggs will be reared as needed to maintain a culture.

5.4.1 (e):

Specify any special needs, such as special handling or housing, that your animals require. (Guide p 28)

Offspring will be housed in aquaria, outdoor mesocosms, and AHAB units similar to those used for adults.

5.4.1 (f):

Describe how you will identify your animals. (Guide, p 25 AW Regs 9 CFR Part 2, sec 2.31)

Sexes of individual Medaka adults will be identified based on physical appearance (anal fin and abdomen shape)

5.4.1 (g):

Will the animals be typed for the gene of interest?
(e.g. blood, saliva, oral swab, ear punch, or tail snip) (Guide, p 25 AW Regs 9 CFR Part 2, sec 2.31)
Yes No

5.4.1 (h):

What is the age range of the animals to be bred? (Guide, p 25 AW Regs 9 CFR Part 2, sec 2.31)

Sexually mature fish will be bred to maintain stock for experiments. We will use fish between 2-6 months old for optimal breeding.

5.4.1 (i):

Describe the female:male ratio to be used. (Guide, p 25 AW Regs 9 CFR Part 2, sec 2.31)

We will use a combination of 3 females to 2 males, or 2 females to one male ratios.

5.4.1 (j):

For rodents, IACUC policy permits only 1 litter per standard size mouse cage, with weaning at 21 days. Describe how you will prevent more than 1 litter per cage and overcrowding of cages. (Guide, p 56)

5.4.1 (k):

For rats and mice, the standard weaning age is 21 days. If weaning older than 21 days please provide justification below: (Guide, p 56)

Section 6: Animal Use Attributes and Justification

6.1 : Will any technique be performed which would result in loss of sensation or paralysis in conscious animals? (Guide, p 123 USDA Animal Care Resource Guide Policy #11)
Yes No

6.2 : Will any technique be performed which will involve prolonged physical restraint (>30 minutes) in unanesthetized animals
other than routine caging and handling? (Guide, p 29)
Yes No

6.3 : Will any agents, such as Complete Freund's Adjuvant or other adjuvants be injected which could cause chronic inflammation and/or pain? (Guide, p 27 USDA Animal Care Resource Guide Policy # 11)
Yes No

6.4 : Will animals be subjected to potentially painful procedures for identification, e.g. toe clipping, branding, etc? (Guide, p 25 USDA Animal Care Resource Guide Policy # 11)
Yes No

6.5 : Will it be necessary for live animals to be removed from the animal facility? (This includes any rooms outside the animal facility even if it is in the same building.) (Guide, p 25-26)
Yes No

6.6 : Will animals be subjected to more than one major survival surgical procedure? (Guide, p 30 AW Regs 9 CFR Part 2, sec 2.31 USDA Animal Care Resource Guide Policy # 14)

Major survival surgery is defined as the penetration of a body cavity with anything larger than a needle or a surgical procedure that results in the permanent impairment of physical or physiological functions.

Yes No

6.7 : Will this experiment involve the study of stress, pain, or abnormal behavior in live animals which cannot be alleviated with drugs because their use would interfere with the research goal? (US Government Principles, Principle # 4, 5 Guide, p 27 USDA Animal Care Resource Guide Policies # 11, 12)
Yes No

6.8 : Do you expect any adverse effects on animals or overt signs of illness as the result of any procedures or activities performed? If YES, explain.
Do not include monitoring intervals or humane endpoints here - monitoring and humane endpoints should only be listed in sections 11.2 and 11.3, respectively to avoid inconsistencies. (Guide, p 105)
Yes No

6.9 : Exceptions with Scientific Justification / Special Husbandry Requirements:
Please specify any exceptions, such as single housing of social animals, husbandry provided by PI or PI staff, or other special husbandry requirements. Provide a brief justification for each item: (US Government Principles, Principle # 7 Guide, p 25-26, Chapter 3 AW Regs 9 CFR Part 2, sec 2.31)

Husbandry Requirement / Exception: Choose a Husbandry Requirement...
Justification:

6.10 : Environmental Enrichment: Environmental enrichment is provided to all confined research animals in accord with the

UGA Environmental Enrichment Policy. Exemption from this enrichment plan requires scientific justification.

Does this project require exemption from the UGA Environmental Enrichment Policy? (Guide p 25-26, 52)

Yes No

6.11 : Food/Water Restriction

Will food or fluid be restricted? (Guide, p 30 USDA Animal Care Resource Guide Policy # 11)

Note: This does NOT include fasting before anesthesia -- please describe fasting before anesthesia in section 9 (if surgery is being performed) or in 8.1/8.2 (if surgery is not being performed). For species that are not fed daily, do NOT include withholding food if the period of withholding is known to fall within that species' normal feeding intervals (e.g., a couple days for reptiles).

Yes No

6.12 : Numbers and Use Category Summary:

The following is an outline of the species selected in section 5.1 grouped by species/highest use category. This represents the number of animals available at the specified use category for animal orders over the 3 year AUP lifespan. If modifications are necessary please make appropriate changes to the species information in section 5.1.

If you wish to increase these numbers after this AUP is approved an amendment will need to be submitted.

HIGHEST USE CATEGORY: A

Species: FISH **Use Category: A** **Number of Animals: 6000**

Section 7: Alternatives Literature Search

7.4 Narrative on Duplication: Please provide a written assurance that the proposed work is not unnecessarily duplicative. The assurance should include a brief narrative on why this work is not unnecessarily duplicative; what makes it novel research or a necessary duplication? (Guide, p 25-26 AW Regs 9 CFR Part 2, sec 2.31)

There are presently only 2-3 research articles utilyzing proteomics approaches to high level radiation exposure on model species, Medaka. However, there are few to none on low level doses which can have substantially different biological responses. Therefore, it is vital to assess the potential harmful effects chronic, low doses of radiation has on cellular processes such as protein expression and function. We have the unique opportunity to combine the SREL LoDIF facilities with the Complex Carbohydrate Research Center (CCRC) to examine this research question. This research group has a long standing reputation for performing current mass spectometry methods to analyze protein function.

Section 8: Experimental Design and Procedures

8.1 : Experimental Design and Procedures: (Guide, p 25 AW Regs 9 CFR Part 2, sec 2.31)
Identify and briefly describe each procedure that will be used in this project. Procedure outlines for inclusion in your Animal Use Protocol are available for several common procedures at https://research.uga.edu/oacu/procedures/. These procedures (denoted by an asterisk in the dropdown list below) are endorsed by the IACUC and may be cut and pasted directly into the protocol. Ensure you copy the entire procedure including the title. Be sure to use the procedures as described under "Artemis Briefs: Animal Use Protocol Descriptions," because they have the correct wording (verb tense) for an AUP.

Please consult the URAR veterinarians regarding any changes made to the procedures.

In-depth detailed descriptions for each procedure are also available at https://research.uga.edu/oacu/procedures/ Note that these descriptions should not be copy/pasted into your AUP because they do not have the correct wording; they are instructional.

Procedure: Anesthesia
Description:

Medaka fish (eggs, juveniles, and adults) will be reared and a culture maintained using standard aquaculture methods at the SREL Par Pond Laboratory. Experimental Medaka will be exposed to varying levels of irridiation in outdoor mesocosm at the SREL LoDIF. Medaka fish will be anesthesized using buffered tricaine methane sulfonate MS222 only at SREL.

8.2 : Description of Procedures: (Guide, p 25 AW Regs 9 CFR Part 2, sec 2.31)

Describe the use of animals in your project in detail. Use terminology that will be understood by individuals outside your field of expertise.

Each experiment/study described in this section must be answered in 3 parts:
Part A: Hypothesis, Part B: Experimental Design and Part C: Experimental Procedures.
Notes: For instructional protocols, you can answer Parts A and B as Not Applicable.
If multiple studies/objectives are described, address Parts A-C for each study before describing the next.

Part A: Hypothesis
Please clearly state the experimental hypothesis and specifically explain how your experiment is designed to test the hypothesis:

- How will you capture the variance?
- What data are you collecting to analyze?
- What conclusions could be drawn from the data if generated as planned?

Part B: Experimental Design

- Clearly define the experimental design, listing each experimental/control group, and the number of animals per group
- You must address specifically how the sample size was determined to be appropriate to capture the variance and test the hypothesis. Please include any assumptions regarding variability, distribution, etc.

Part C: Experimental Procedures

- Provide a detailed description of all animal procedures in a logical, chronological progression, beginning with receipt of the animals and ending with the study endpoint.
- Specific procedures, test, analyses, treatments, assays, drugs/compounds, duration of the study/timeline, and point at which animals are removed from the study (e.g., euthanized) should be clearly delineated for treatment and control groups.
- You must address specifically what data is being collected
- **Surgical procedures and anesthesia details should be entered into sections 9 and 10, respectively. Surgery and anesthesia details should not be entered here in 8.2, to avoid inconsistencies.**
- **Note that you should not include doses/volumes here for drugs listed in section 10 -you should only list the doses in section 10, to avoid inconsistencies**

Par Pond Laboratory/LoDIF Medaka Husbandry
Standard Operating Procedures

I. Purpose: This SOP outlines a simple and humane method to rear Japanese Medaka fish, Oryzias laticeps, in aquatic housing systems, standard aquaria, and in outdoor mesocosms used for experiments at the University of Georgia's Savannah River Ecology Laboratory (Facility location 737-G). These Pentair Aquatic Habitats (AHAB's) are one of the most widely utilized systems for aquatic research animals in the world, providing a flow through system with reverse osmosis, bio filtration, and UV sterilization.

II. Scope: The described procedure is applicable to Medaka fish, Oryzias laticeps used for maintaining cultures of fish for experimental purposes at the University of Georgia's Savannah River Ecology Laboratory located in Aiken, South Carolina.

III. Equipment & Supplies.

A. The necessary number of indoor 1.4 and 2.5 liter AHAB tanks in 1-5 separate AHAB systems as recommended by the manufacture (Pentair) and required for experimental design and maintaining animals. Standard 10 gallon aquaria will also be used for housing aquatic organisms.

B. Appropriate equipment for water quality monitoring (Ph, temperature, nitrate, etc.).

C. Source of food: a combination of brine shrimp, dry flake, or black worms.

D. The necessary number of five gallon buckets modified with mesh screens to allow for flow through while experiments are conducted in mesocosms at the Low Dose Irradiation facility during experimental trials.

E. Anesthetic solutions and liquid nitrogen for preservation of tissue for proteomics.

IV. Introduction: The Savannah River Ecology Laboratory's Par Pond Facility has been used to successfully perform experiments on a variety of aquatic organisms since 2002. Aquatic organisms are maintained in either an indoor laboratory or outdoor mesocosms during experiments. Pentair aquatic habitats (AHAB') are widely used to rear fish for embryological, toxicological, and genomic studies. The LoDif is an outdoor, gamma-irradiation array consisting of 40 fiberglass, open-air tanks designed to house a variety of aquatic organisms. Each tank, or mesocosm, is parabolic in shape, 2.4 m in diameter and holds approximately 965 L of water with a maximum depth of 41 cm. Mesocosms are of a flow- through design and receive water from a nearby lake at rates that are adjustable to about 1L/min. The use of outdoor mesocosms containing buckets of aquatic organisms allows for the controlled design of replicated radiation dose studies in this facility. For randomized block experimental designs, the facility is arranged into eight 6.5m X 10.5 m blocks, with each block containing five mesocosms. This facility, in addition to an indoor aquatic rearing laboratory can provide great insight into the effects of chronic low dose irradiation across 4 levels, control (indoor and outdoor), low (.74MBq), medium (7.4MBq), and high (74.0MBq) doses corresponding to multiple independent treatments (Hinton et al. 2004). Some animals will be exposed to varying levels of radiation using the 137-cesium gamma sources at the LoDIF, and others (control AHAB and control LoDIF, culture stocks) will not be exposed to radiation.

V. Cited Literature

Ding, L, Kuhne, W.W., Hinton, D.E., Song, J., and Dynan, W.S. 2010. Quantifiable Biomarkers of Normal aging in the Japanese Medaka Fish (Oryzias laticeps). PLoS ONE 5: e13287. Doi:10.1371/journal.pone.0013287

Kinoshita, M., Murata, K., Naruse, K., and Tanaka, M. 2012. Medaka: Biology, Management, and Experimental Protocols. Wiley-Blackwell Publishing, Iowa.

Hawkins, W.E., Walker, W.W., Fournie, J.W., Manning, C.S., and Krol, R.M. 2003. Use of the Japanese Medaka (Oryzias latipes) and Guppy (Poecilia reticulata) in carcinogenesis testing under national toxicology program protocols. Toxicologic Pathology 31: 88-91.

Hinton, T.G., Coughlin, D.P., Yi, Y., and Marsh, L.C. 2004. Low dose rate irradiation facility: initial study on chronic exposures to medaka. Journal of Environmental Radioactivity 74: 43-55.

Leary et al. 2013. AVMA guidelines for the Euthanasia of animals: 2013 edition. American Veterinary Medical Association. Available: https://www.avma.org/KB/Policies/Documents/euthanasia.pdf

Yamamoto, T. 1975. Medaka (Killifish) Biology and Strains. Keigaku Publishing, Tokyo.

IV. Animal care (Par Pond Laboratory Medaka Husbandry)

*Amount of Medaka needed: We are requesting to maintain 2,000 fish annually over the next three years. Each experiment conducted at the LoDIF requires ~576 fish under full use (12 fish per bucket * 6 treatments (1 High, 1 Medium, 1 Low exposure, 2 outdoor controls, 1 indoor control) * 8 blocks). We expect to be performing multiple experiments over the next three years as well as maintaining a minimal number of adult and juvenile fish on hand for culturing purposes in multiple AHAB's. We will need to maintain laboratory cultures, multigenerational lines of experimental fish for ongoing research, as well as fish for subsequent experiments as dictated by research needs. Each experiment may consist of up to ~576 adults exposed for several weeks at the LoDIF. We may also perform additional experiments on ~576 juveniles, or ~576 eggs as determined by the results of our preliminary experimental approach. Because Medaka life span under laboratory conditions can vary from ~12 months to 22 months (Ding et al. 2010, Hawkins et al. 2013), we will be housing fish at various life stages (eggs, juveniles, adults) over the course of three years in order to replace aging fish, maintain breeding pairs, and have an adequate number of fish for experimental purposes. Therefore, we expect to perform 2 full experiments per year (~1,200 fish) and maintain a culture (~800 fish/year) as needed.*

Species: Medaka will be maintained at the Par Pond facility under appropriate conditions for experimental trials at the Low dose irradiation facility (LoDIF). Medaka fish (Oryzias laticeps) will be housed indoors and maintained for outdoor experiments. Wild type Medaka will be purchased from either a distributor in Japan, Aquatic Ecosystems Inc., or provided by Dr. Kullman at North Carolina State University. Medaka have served as an experimental model since the early 1900's (Yamamoto 1975) and are one of the most widely used fish species for embryological, toxicological, and genomic studies. This species has numerous advantages for experimental laboratory and field studies, including tolerance for a wide variety of water conditions, small size (average length of 25 to 50 mm and weight ~0.7 to 0.8 grams), short generation time (~6 to 8 weeks), and prolific capacity to reproduce (6 to 30 eggs per day; 3,000 eggs per female/breeding cycle and males capable of fertilizing eggs from several females (Yamamoto 1975, Kinoshita et al. 2012).

Record keeping: A water quality data sheet will be maintained daily (for daily checking) for each unit/tank to record water parameters (e.g., temperature, pH) daily. We will also monitor individuals within AHAB's used for culture/experiments. Fish health (physical appearance) will be monitored daily.

Room condition: The temperature of the Par Pond Laboratory will be maintained between 25 and 28C using air-conditioners and/or tank heaters with an automatic thermostat. Windows of the facility allow for some natural light penetration.

Acclimation: Prior to setting up new aquaria, tanks will be washed and rinsed thoroughly with Simple Green nontoxic cleanser. Tanks will be filled with dechlorinated water and aerated for a minimum of 24 hours prior to adding fish. When using heaters, we will allow the units to acclimate in tanks for at least 15 minutes before plugging them in. Water conditions will be monitored for at least 24 hours before adding fish.

Upon arrival, fish shipments will be examined and any dead animals immediately removed. If the temperature between tanks and shipping/collection water is within 2°C, fish will be carefully netted into new tanks. If there is more than a 2°C difference between tanks and shipping/collection water, we will allow sealed bags to soak in the new tanks water until the temperatures are within 2°.
C. Fish will then be transferred into new tanks.

Containers: Glass containers of 50X25 cm in size, holding ten gallons of water will be used for indoor culture stocking. About 30 adult fish will be housed in each aquarium equipped with aerators, bio-filters and ammonia extracting bags. For stocking density, we will use ~25 sq. cm per adult fish as the spatial requirement. In addition pairs will be kept in Pentair Aquatic habitats© Z rack systems (AHAB). These units are specifically designed to house small aquatic species for experimental/laboratory research. Medaka will be kept in small 1.4 liter AHAB tanks (mating pair) or large 2.5 liter AHAB tanks (up to 10 fish). AHAB small tanks with a volume of 1.4 L (length=21 cm, width 4cm, area= 84sq cm) will house 2 fish with a surface area of 1 fish per 42 sq cm in a rearing density of .35 g in. 7 liter of water. Large AHAB tanks with a volume of 2.5 L (length=21 cm, width= 8 cm, area=168 sq cm) will house up to 6 fish with a surface area of 1 fish per 5 sq cm and a rearing density of 0.35 g in .7 L of water. All tanks will be labeled accordingly as needed for maintaining either culture/experimental adults, juveniles, or eggs.

Water Temperature/Level: Medaka will be maintained at 22-24°C while non-breeding and at 25~26°C while breeding within aquaria and AHAB systems. Water levels will be checked daily and periodically adding unfiltered PAR pond water as necessary due to the loss of water by evaporation. Water levels will be checked daily. For partial water changes, tank water will be siphoned into buckets, checking each tank and bucket for fish individuals before discarding water and replacing.

Nitrite/Ammonia: Ammonia and nitrite levels will be monitored daily and maintained at zero level (below 1.0 ppm for ammonia and 0.1 ppm for nitrite). A 50% water change will be performed biweekly, or as needed regardless of zero levels in ammonia and nitrite. A total water change will be performed if nitrile levels are elevated or any fish within the container become ill.

pH: PH of water will be measured daily to ensure that it is between pH 6 and pH 7 with a YSI water quality probe or standard aquarium pH testing kit. If a ph value is outside this range, a total water change of up to ~50% will be made. A small amount of algae will be allowed to grow on tank containers. The surfaces of tanks will occasionally be cleaned with a small piece of filter mat (plastic wool) to maintain a clear view within the tank. Bio-filters and ammonia extract bags will be periodically washed and cleaned with pond water. Tanks will be held between 22-25 °C with tank heaters.

Photo Period: A light-dark cycle will be set by adjustable room timers for 14 hrs. light and 10 hrs. dark. The Par Pond Laboratory does allow some amount of natural light.

Food: Fish will be fed two times a day with live brine shrimp (Artemia sp.) or Aquatox diet dry flake. Brine shrimp and dry flake are both highly suitable feed for Medaka larvae, juveniles, and adults (Kinoshita et al. 2012). Fish may also be fed black worms. Quantity of food should be 4-5 % of the body weight. If necessary, we will finely grind a dry food mixture to be used in auto-feeders at weekends. Care will be taken to remove and clean the dry food stuff remains floating on the surface or sticking to the bottom of the fish habitat. Food ration quantity or frequency may be increased to 10% of body weight during breeding (up to 5 times a day, typically with 3 rations of brine and 2 dry flake feedings).

NON-breeding Adult/Juvenile Care: When in non-breeding mode, Medaka will be maintained in either 10 gallon tanks at a maximum density of 30 fish per tank, small 1.4 L AHAB tanks (2-3 fish), or large 2.5 L AHAB tanks (6 fish). AHAB tanks have a continuous source of water intake in a flow through system while standard 10 gallon aquarium tanks will be equipped with water filtration systems (aerators, bio filtration and ammonia extraction bags). Fish will be fed at least once per day, typically twice per day, with live brine shrimp ensuring the quantity of food to be 4-5 % of the body weight (U.S. EPA).This generally corresponds to ~1/8 of a teaspoon of finely ground food for a ten gallon tank with 30 fish. If necessary, a dry food mixture will be used in auto-feeders on weekends. Care will be taken to periodically remove and clean the dry food stuff remains which float on the surface or stick to the bottom. Tanks will be examined for emaciated, deformed, or diseased fish. Any dead fish will be saved in a vial and labeled with all relevant information and placed in a freezer.

Breeding Adult Care: We will maintain a culture of Medaka as needed for experimental purposes at the Savannah River Ecology Laboratory. For breeding, we will use 2.5 liter AHAB flow through tank systems and glass containers 50X25 cm in size holding ten gallons of water for indoor culture stocking. For stocking density, we will use ~40 sq. cm per adult fish as the spatial requirement. Medaka exhibit sexual dimorphism; the male has a leger anal fin and a dorsal fin that is deeply notched between the two rays closest to the body. Breeding females are distinguished by their distended abdomens. Medaka sex ratio for broodstock will consist of one male: two female or two male: three female pairs. Water temperature in the breeding mode will be 25-26°C. Photoperiod will be set according to season, typically to 14 hrs. light and 10 hrs. dark to mimic natural lighting. Ammonia levels, nitrite levels and pH of water within tanks and containers will be monitored daily. Water levels in reservoir sumps will be checked daily. When new water is added to compensate for evaporation, temperature adjustments will be made, allowing for only a 2°C difference in new water to existing water. Feeding time will be increased from one to 3-5 times per day. Under these conditions, females will be producing eggs in about 10-14 days. Generally, female Medaka produce eggs within 1-2 hours after the lights have turned on and one female Medaka normally produces 10-30 eggs per day. Female fish carry eggs in the abdomen in front of the anal fin.

Egg Care and Collection: For the collection of eggs, we will turn off water in each individual tank and transfer all contents of each AHAB tank to a secondary container, accounting for all eggs. We will separate adult pairs from eggs by using a fine fish net and gently brush off hanging eggs from the adult female fish abdominal area if necessary. Both adult fish will be promptly returned to the proper tank to prevent further stress. The original tank will be checked once more for any additional eggs which may have been laid on the bottom of the tank. Eggs will be cleaned using a mesh screen to remove the filament that binds the cluster of eggs or by the finger sorting method (rubbing eggs using a forefinger in a Petri dish; Kinoshita et al. 2012). Eggs from each pair will be reared in separate tanks in dechlorinated water or laboratory grade embryo rearing solution (Carolina Biological Supply) until hatching. A Methylene Blue solution (0.00001%) may be used to prevent fungal infections or water mold (Saprolegnia sp.) and will be prepared as recommended or as needed. Dead eggs will be removed daily with small metal forceps or disposable pipettes to prevent fungus. Ethanol will be used to clean forceps and prevent cross contamination.

We will transfer hatchlings into the appropriate container (small plastic Tupperware containers) with the help of a disposable pipette. After the first 24-48 hrs. hatchlings will be fed live brine shrimp nauplii, once per day. Hatchlings will be fed twice per day once they are one week old. Mortality of eggs will be checked daily and tanks housing eggs will be monitored in a similar manner as adults. Juveniles will be housed in either AHAB systems or aquaria for maintaining/replacing experimental stock of Medaka. Care will be taken to house fish in separate tanks according to life stage / fish body size to prevent adults from eating larvae (Kinoshita et al. 2012).

Transportation of animals: Animals used for experimental trials in outdoor mesocosms (LoDIF) will be transported between the LoDIF and Par Pond lab (~1 minute walk) in 5 gallon buckets. Tissue samples f or Proteomic analysis will be made either on site or at the Complex Carbohydrate Research Center on the University of Georgia campus. No live animals will be transported to the CCRC on UGA campus. All Medaka will be euthanized at SREL and only tissue from deceased fish will be transported to the CCRC.

Euthanasia of animals: Animals sacrificed for proteomic tissue research (or sick diseased fish) will be euthanatized by an overdose via immersion in anesthetic solution of MS-222/tricaine methane sulfonate buffered with sodium bicarbonate at a minimal concentration of 250mg/l at or near neutral pH.
A concentration of 250-500 mg/L (5-10 times the anesthetic dosage is effective for Medaka according to AVMA 2013 guidelines (Leary et al. 2013). Medaka will be left in the anesthetic solution for a minimum of 10 minutes after cessation of opercular movement. Tissues used for the Radiation/Proteomics study will be frozen in liquid nitrogen and stored at -80 C until extracted for Proteomics analysis. Euthanasia of animals will occur only at the Savannah River Ecology Laboratory. Only tissue samples from deceased fish will be brought to the CCRC on UGA's campus for mass spectrometry analyses. No live fish will be brought to the UGA CCRC.

VII. Animal Care (LoDIF irradiation experiments)

Overview: Medaka will be transferred from the Par Pond laboratory to the LoDIF for experimental trials at varying exposure rates. The LoDIF (694-G) is located at coordinates 3680754, 449260 UTM, adjacent to The Par Pond Radioecology Lab (SRS building 737-G), and is within a designated radiological Controlled Area. The facility is approximately one acre in size and is enclosed by an eight-foot chain link fence. The fence is reinforced along the bottom with partially buried hardware cloth to prevent intrusion by animals. This perimeter fence has two locked gates, one personnel gate on the west end and one vehicle access gate on the south side. The 8-foot fence is posted as requiring personnel dosimeters and a Radiological Work Permit to enter. Keys to the perimeter gates are under positive control by the facility custodian. The keys are stored within a lock-box in the facility custodian's office (737-G, Room 3). The lock-box is used exclusively for keys pertaining to the Low Dose Irradiation Facility (i.e. perimeter gates, inner pad gates, and individual radiator locks).

Exposure Methods: One of SREL's primary contributions to the proposed research is a Low Dose Rate Irradiation Facility (LoDIF). The LoDIF is an outdoor, gamma-irradiation array consisting of 40 fiberglass, open air tanks designed to house a variety of aquatic organisms covering an area of 0.4 ha in area. This unique research facility can deliver chronic, low dose radiation treatments to any of the 40 mesocosms. Specially designed irradiators are mounted on steel frames and placed over each mesocosm. Each irradiator contains a 0.74, 7.4, or 74.0 MBq sealed 137Cs source within a lead container collimated to deliver an exposure to animals within modified buckets with mesh screens residing in tanks below. A wide spectrum of dose rates is achievable within the facility, ranging from < 0.1 mGyd-1 to ~250 mGyd-

1. Medaka within mesocosms are externally irradiated and as to not become contaminated with radioactive material. Hence Medaka/tissue samples can be sent to clean laboratories for various analyses. Thermoluminescent dosimeters, placed within the exposure field, obtain accurate dose rate estimates. The precision that dose rates can be estimated within the facility reduces the uncertainties often associated with dose-response relationships. A blocked, randomized design utilizing the structural capacity of the LoDIF allows for replicated, powerful statistical analyses.

Acclimation: Animals transferred to the outdoor LoDIF from the Par Pond Laboratory will be acclimated to external temperatures by remaining in buckets long enough to allow for a + 2 °C water difference. Water replacements will be made to buckets as needed before placing buckets within mesocosms.

Containers: Medaka will be maintained during the course of experiments (several days up to several weeks) in modified 5 gallon buckets with mesh to maintain water levels. These buckets will be placed within larger mesocosms directly under sealed sources or controls. This outdoor experimental design allows for placing one to 4 buckets within each mesocosm. Outdoor tanks will be cleaned and water levels maintained prior to placing any fish at the LoDIF.

Water levels: Water for individual mesocosms is pumped directly from Par Pond. Water levels will be checked daily and replaced as needed to compensate for evaporation. A maximum of 12 Medaka adults will be placed in each 5 gallon bucket.

Food: Fish will be fed daily by placing a premeasured amount of flake/brine shrimp in a modified extension pole with a feeding cup and poured into each individual 5 gallon bucket. The amount of food will be determined by the number of fish within each bucket and follow our rearing protocols. While in the outdoor mesocosms, fish diet will be supplemented by invertebrates that periodically fall into these tanks.

Record Keeping: The number of live fish in each bucket, and fish health (physical appearance) will be monitored daily during experiments in the LoDIF. Datasheets will be maintained daily (for daily checking).

Experimental Animal Euthanasia: Animals used in LoDIF experiments will be returned to the Par Pond Laboratory and either euthanized for Proteomics analysis, or cultured within AHAB systems for future study. Animals sacrificed for proteomic tissue research (or sick diseased fish) will be euthanatized by an overdose via immersion in anesthetic solution of MS-222/tricaine methane sulfonate buffered with sodium bicarbonate at a minimal concentration of 250mg/l at or near neutral pH. Euthanasia will only occur at SREL.

8.2.1: Please describe the proposed procedures impact on the animals well being. (Guide, p 25) Note that this question **must** be answered, even if no impact is expected.

Medaka exposed to radiation may experience subclinical effects as a result of exposure, which will not make them ill. Previous studies on Medaka at these exposure levels found primarily subclinical effects for control, low, medium, and high exposure levels. However, Medaka exposed at our highest dose level may experience a limited number of adverse effects including cellular mutation and reduced reproductive success, but no clinical illness problems from irradiation have been noted. Medaka have been used successfully for experiments at the LoDIF at these low dose radiation levels for several years.

Animals will be maintained following standard aquaculture techniques. We will follow approved protocols to minimize distress of experimental/culture animals.

8.3 : Will you be using potentially hazardous substances in live animals? These potentially hazardous substances include chemicals, biohazards (including human or animal cell lines), recombinant DNA, or radiation. (Guide p 18, 25-26 Occ Health and Safety, p13)

(●) Yes ( ) No

8.3.1 (a):

Select the category of hazard that will be used and then provide the name, the species in which it will be used, a short description of its use and what methods will be employed to ensure it is used safely, and a description of all Personal Protective Equipment (PPE).
Note that the safety methods described should focus on the use of the hazard in the animal facility/housing location, not the preparation in your lab. You must explain how soiled bedding is dumped when cages are changed

and what disposal method will be used for the soiled bedding and carcasses. Note that you must notify URAR in writing before a hazard is administered to animals and that all cages in which such animals are housed must be labeled with a URAR approved label.

- [ ] Hazardous Chemicals:
- [ ] Biohazards and rDNA:
- [x] Radiation:

Description of use in animals and procedures to ensure safety of personnel including PPE as recommended by the Office of Radiation Safety:

Radioactive Substance:	*Cesium 137*
Species Used:	Fish
Do you have all appropriate permits to work with this substance?	○ Yes ◉ No
Description of use and procedures to ensure safety of personnel recommended by the Office of Radiation Safety:<br><br>*A centerpiece of SREL's radioecology capabilities is the Low Dose Irradiation Facility (LoDIF), which is used to study the e¬ffects of low-level, chronic irradition on biota. The LoDIF is an outdoor gamma- irradiation array consisting of 40 fiberglass open-air tanks (experimental mesocosms) in a secure area. Dose rates are known for organisms housed in buckets below each irradiator. SREL has worked through DOE to acquire all neccessary permits for the facility on the SRS; workers who enter the facility are all required to have specialized rad worker training*	

Section 9: Surgical Information

9.1.1: Surgical Information (AW Regs 9 CFR Part 2, sec 2.31 Guide p 25-26, 105, 115)
Will you be doing surgery?
NOTE: Anesthetizing an animal for terminal tissue collection before the animal is euthanized is considered Non Survival surgery and should be included in section 9.
○ Yes ◉ No

Section 10: Administration of Anesthesia, Analgesia, Other Substances and Biologicals

10.1 : Will you be using anesthesia, anesthetic antagonists, analgesics, tranquilizers, etc.? (Guide, p 12, 25, 105, 121 AW Regs 9 CFR Part 2, sec 3.21 USDA Animal Care Resource Guide Policy # 3)
◉ Yes ○ No

10.1.1 (a):

List all **pre-anesthetic agents** (e.g., tranquilizers, narcotics), **anesthetic agents, antagonists** and **analgesics** and describe how these agents will be used in your studies.

Agent	Tricaine methanesulfonate (MS-222)
Frequency of Anesthesia	*once for tissue collection/diseased animals*
Species	Fish
Dose	*1X-5X*
Route	Immersion
Volume	*250-500 mg/L*

10.1.1 (b):

Monitoring of Anesthesia (Guide, p 12, 119 USDA Animal Care Resource Guide Policy # 3)
Check all items that you will be monitoring and provide a brief description of the monitoring, depth assessment, thermal support, and recovery, in the text box below.

Include:

- How frequently each of these variables will be monitored. Note that some, such as respiration rate, should be monitored continuously.
- How you determine that the depth of anesthesia is adequate before a painful procedure is performed (e.g., lack of response to a firm toe pinch).
- The method of thermal support that will be provided. Please note that the use of an electric heating pad requires a barrier between the animal and the pad (e.g., a folded towel underneath the recovery cage).
- Monitoring during the immediate recovery from anesthesia. Note that an animal must be monitored continuously until it is able to hold itself in a normal, upright position.

☐ Body Temp ☐ Pulse Oximetry ☐ Blood Gas ☐ Doppler Blood Flow ☐ Mucus Membrane Color
☐ Capillary Refill Time ☐ ECG ☐ Blood Pressure ☐ Respiration Rate ☐ Capnography ☐ Muscle Tone

Euthanasia is the expected outcome. See Section 8.2

10.1.1 (c):

Provide a plan for immediate (<48 hours) and extended (>48 hours) post-surgical analgesia: (Guide, p 12, 25, 105, 120 AW Regs 9 CFR Part 2, sec 2.31 USDA Animal Care Resource Guide Policy # 3)
Include:

- The drug/s to be administered, and the frequency at which they are administered.
- Be sure to distinguish the standard analgesia plan from additional analgesia that will be provided "if needed." In other words, what is the standard plan, that all animals will receive, and what will be done if an animal is still showing signs of pain after the standard plan has been completed?

Euthanasia is the expected outcome. See Section 8.2

10.1.1 (d):

Describe how animals will be assessed to determine the need for analgesia after a surgical procedure: (Guide, p 120)

Include:

- What signs will be monitored?
- How often will animals be monitored? Note that animals should be assessed for the need for additional analgesia at time intervals appropriate to the analgesic being used. I.e., if the analgesic lasts for 6-8 hours, the animals should be reassessed 6-8 hours after the previous dose.

Euthanasia is the expected outcome. See Section 8.2

10.2 : Will you be administering substances to live animals other than anesthetics, analgesics, etc. listed in 10.1?
(This would include any therapeutic drugs; cells or tissue extracts and experimental or study materials such as infectious agents, antigens, adjuvants, or other reagents) (Guide, p 12 AW Regs 9 CFR Part 2, sec 2.31)

○ Yes ◉ No

10.3 : Are non-pharmaceutical-grade chemicals or other substances used? (Does not apply to test articles) (Guide, p 12, 31 USDA Animal Care Resource Guide Policy # 3)

○ Yes ◉ No

Section 11: Monitoring

11.1 : Monitoring Interval: (Guide, p 12, 25-26)

Indicate, by checking one or more of the appropriate boxes below, the minimum frequency with which your lab personnel will monitor your animals during and after all procedures. If more than one frequency will be followed (i.e., different studies, different time periods during the study), please check all of the appropriate boxes.

- This question does not refer to the standard daily observations made by University Research Animal Resources staff.
- Note that for USDA covered species, this monitoring by lab personnel for signs of pain/distress related to the research procedures must be documented in the clinical health record.

"Approximately" below indicates within the range of 1 before to 1 hour after the time stated.

☑ Daily

☐ Approximately every 6 Hours ☐ Approximately every 8 Hours ☐ Approximately every 12 Hours ☐ Approximately every 24 Hours

☐ Other

11.2 : Provide a brief description of the monitoring interval checked above. (Guide, p 12, 26)
NOTE: THE MONITORING DESCRIBED IN 11.2 SUPERCEDES DESCRIPTIONS IN OTHER SECTIONS IF OTHER SECTIONS ARE NOT CONSISTENT WITH 11.2. THE IACUC'S EXPECTATION IS THAT THE MONITORING SCHEDULE IN 11.2 WILL BE FOLLOWED.

- If the frequency will vary during the experiment (e.g., the time from injection to tumor visible to the time of euthanasia) describe all periods of time.
- Note that the interval between checks should include a specific hour range (i.e., "every 10-12 hours" or "once between 7 to 9 am and a second check between 6-8pm" not "twice a day")
- If the frequency will vary by experimental group (e.g., animals infected with different strain of virus) describe the frequency to be used for each group.
- If parameters are being measured to determine the humane endpoints (e.g., weight, tumor size) include the frequency at which the measurements will occur.

Adult/juveniles and embryos used for experiments/culture will be monitored daily for survival/health. Water conditions will be checked daily as stated in the attatched operating procedure (section 8.2). Dead embryos/diseased fish will be removed when discovered.

Section 12: Animal Disposition

12.1 : Final Disposition (Guide, 26, 105, 123)

For each species listed on this AUP, describe what will happen to these animals at the end of this project:
By default, PI retains the right to transfer animals to other protocols at the discretion of the attending veterinarian and in accordance with UGA policy.
Please note that while euthanasia may not be a planned outcome of the study, a method of euthanasia must be described (question 12.2) should an animal experience unanticipated pain or distress (e.g. untreatable injury or illness).

Fish

☐ Planned transfer to other projects (Transfer of animals to a different PI or to a different AUP must be handled through URAR, via AnOps) (Explain in the text box):

☐ Adoption or resale* (Explain in the text box):
☐ Return to the wild* (Explain in the text box):
☐ Return to the herd or flock* (Explain in the text box):
☑ Euthanasia (Describe the methods in 12.2)

Animals sacrificed for proteomic tissue research (or sick diseased fish) will be euthanatized by an overdose via immersion in MS-222/tricaine methane sulfonate buffered with sodium bicarbonate at a minimal concentration of 250mg/l. Concentration will vary by size and life stage. A concentration of 250-500 mg/L (5-10 times the anesthetic dosage is effective for Medaka according to AVMA 2013 guidelines (Leary et al. 2013). Medaka will be left in the anesthetic solution for a minimum of 10 minutes after cessation of opercular movement. Euthanasia will occur only at SREL.

12.2 : Euthanasia Method (Guide, p 126, 105, 123 AW Regs 9 CFR Part 2, sec 2.31 USDA Animal Care Resource Guide Policy # 3)
Describe the method of euthanasia for each species listed in this AUP:
**Please note that while euthanasia may not be a planned outcome of the study, a method of euthanasia must be described in case an animal experiences unanticipated pain or distress (e.g. untreatable injury or illness).*
**Please note that if the method you are using is not approved by the AVMA Guidelines on Euthanasia, you need to justify the method (Check the "Other" box at the end of 12.2 to provide a text box for the justification).*

Fish

☐ Injectable Drugs: If you are using a drug, you must indicate the name, dose and route of administration of the drug.(Check the "Other" box at the end of 12.2 to provide a text box for this information). NOTE: For wildlife studies, it is the responsibility of the researcher to prevent scavenging animals from consuming carcasses contaminated with drugs that are potentially dangerous.

☐ Carbon Dioxide: CO_2 euthanasia requires a second, physical method of euthanasia to ensure death (Check the "Other" box at the end of 12.2 to provide a text box for the description of the secondary physical method).

☐ Physical Methods: (Cervical dislocation and decapitation of animals requires prior sedation. Justification must be provided if you cannot sedate the animals prior to cervical dislocation or decapitation. Furthermore, if you cannot sedate animals prior tcervical dislocation or decapitation, you are required to describe the training or experience for the persons using this method.) Please provide any justifications along with personnel training and experience in the following box:

☑ Other:

Animals sacrificed for proteomic tissue research (or sick diseased fish) will be euthanatized by an overdose via immersion in MS-222/tricaine methane sulfonate buffered with sodium bicarbonate at a minimal concentration of 250mg/l.
Concentration will vary by size and life stage. A concentration of 250-500 mg/L (5-10 times the anesthetic dosage is effective for Medaka according to AVMA 2013 guidelines (Leary et al. 2013). Medaka will be left in the anesthetic solution for a minimum of 10 minutes after cessation of opercular movement. Euthanasia will occur only at SREL.

Section 13: Permits

13.1: Permits (Guide, p 106 State and federal laws that require permits for collection of certain wild animals) Do you have any permits that may be required for handling, importation, collection or maintenance of animals used in this proposed study?

○ Yes ◉ No

Section 14: Certifications and Submission

I, Olin Rhodes, certify that:

☑ This Protocol provides a complete and accurate description of all proposed uses of live vertebrate animals in this research activity. Any proposed revisions to animal care and use procedures will be promptly forwarded in writing to the IACUC for review and approval prior to implementation.

☑ I agree to abide by all applicable laws, policies and regulations, including the U.S. Animal Welfare Act, the National Research Council Guide for the Care and Use of Laboratory Animals, the Public Health Service Policy on the Humane Care and Use of Laboratory Animals, the FASS Guide for the Care and Use of Agricultural Animals in Research and Teaching, and all UGA policies and procedures regulating the humane use of vertebrate animals in instruction and research.

☑ I will comply with all regulations governing the importation, collection and/or maintenance of wild species, including obtaining permits from all applicable regulatory agencies prior to the acquisition of animals.

☑ I have completed (or agree to complete) training (OACU Training) offered by the Office of Animal Care and Use. I agree to participate in required training at least once every 3 years to assure that I remain current on laws, regulations, guidelines and policies pertaining to the use of animals.

☑ The information in this form agrees with the animal use section of the grant application.

☑ All procedures involving live animals will be performed under my supervision.

- I accept responsibility for ensuring that all personnel working with live vertebrate animals are aware of, and will not deviate from, the IACUC approved procedures outlined in this protocol; that they will adhere to the regulations regarding the humane treatment of animals and that they will receive proper training as required by the IACUC.
- All personnel having direct contact with live vertebrate animals, including myself, have been or will be

trained in humane and scientifically acceptable procedures for animal handling, administration of therapeutic drugs and euthanasia to be used in this project.
- Personnel will be allowed adequate time to obtain necessary training for this project and will not begin any procedures with live animals until they have been successfully trained.
- Listed participants will perform only those procedures for which they have received adequate training.

☑ Extramural or intramural peer review for scientific merit will be conducted before this proposed work is initiated.

☑ I understand that personnel with animal contact will be required to participate in the occupational health and safety program. (OHSP Program) I agree to inform all persons working with animals under my supervision regarding the availability of this program. (Contact the IACUC office if you need additional information.)

PI Approval

Signature of Principal Investigator: <-- Electronically Signed by Olin Rhodes -->

Date: 2013-06-04

The Attending Veterinarian verifies that the elements of this proposal have been assessed regarding the use of appropriate techniques in utilization of animals and that consultation with the PI will occur as necessary to resolve issue to minimize pain and distress.

Signature of Attending Veterinarian: <-- Electronically Signed by Leanne Alworth -->

Date: 2013-06-05

IACUC Member Approval

Signature of IACUC Member: <-- Not Yet Signed by IACUC Member -->

IACUC Chair Approval

Signature of IACUC Chair: <-- Not Yet Signed by IACUC Chair -->

Final Approval

<-- Not Yet Received Final Approval -->

Revision Comments:

2013-05-28 09:33:17 Leanne Alworth wrote:
Thank you for this AUP. Please respond to the following questions via a revision:
[4.1] Please clarify what procedures will occur at SREL and what procedures will occur at CCRC.
[5.4.1(i)]This section describes breeders are being 3F:2M or 2F:1M, while section 8.2 uses 1M:1F or 1M:2F. Please make sure this is consistent.
[8.2] Please revise the statement "...a Water quality data sheet will be used for each unit/tank when any parameter is checked daily." It is important that a data sheet is maintained daily (for daily checking) whether or not water parameters are checked daily.
[8.2] Please explain why 5,000 fish are needed. For example, how many fish per treatment level, and why?
[8.2] Are 'black worms' the same as 'blood worms'?
[8.2] The statement "...fish health (physical appearance)is monitored at least twice a week." gives the impression that fish are not assessed for health daily, as is the requirement. At the end of the section, it states "Animals will be fed/checked daily..." which is appropriate. If there is something specific you will do twice a week, clarify what that is, however the fish's health should be assessed daily.
[8.2.1] This should explain how the fish well being may be affected. Mainly, do you anticipate any clinical illness problems from the irradiation, or will all of the changes at this level be subclinical (ie won't make them ill)?
thank you,LA

2013-06-04 11:01:05 Leanne Alworth wrote:
Please make the personnel changes needed.

Renewals

Renewal Form Number: A2013 05-018-R2

Species: Fish Use Category: Category A Number Used in the Past Year: *600*

Indicate briefly what you have accomplished in the last year with this study.
If your study is a Category C, please consider the following questions and provide responses below:

1. What is the percentage of experimental animals being assessed by humane endpoints that were found dead before reaching an established humane endpoint?
2. Does the monitoring interval established by your lab allow you to collect appropriate data yet identify moribund animals and remove them from the study to minimize suffering?

Within the last year of this study we have successfully maintained a total of 600 Medaka fish at SREL's Par Pond facility as both breeding stock and as experimental fish housed in large outdoor tanks (10 fish/mesocosms) within the LoDIF (Low Dose Irradiation Facility) according to our AUP. We are continually rearing fish in AHAB systems and tanks indoors at the Par Pond Laboratory, so at any given time the laboratory houses Medaka at various life stages (adults, young adults, and developing juveniles from eggs collected). We have also euthanized 12 fish at SREL for conducting preliminary proteomic tissue analysis following our AUP . No modifications of our animal use proposal are currently needed.

PI Approval

Signature of Principal Investigator: <-- Electronically Signed by Olin Rhodes -->

Date: 2014-03-17

The Attending Veterinarian verifies that the elements of this proposal have been assessed regarding the use of appropriate techniques in utilization of animals and that consultation with the PI will occur as necessary to resolve issue to minimize pain and distress.

Signature of Attending Veterinarian: <-- Electronically Signed by Leanne Alworth -->

Date: 2014-03-17

Final Approval

Received Final Approval on: 2014-03-26

Revision Comments:

No Revision Comments made for this Renewal.

Renewal Form Number: A2013 05-018-R4

Species: Fish Use Category: Category A Number Used in the Past Year: *500*

Indicate briefly what you have accomplished in the last year with this study.
If your study is a Category C, please consider the following questions and provide responses below:

1. What is the percentage of experimental animals being assessed by humane endpoints that were found dead before reaching an established humane endpoint?
2. Does the monitoring interval established by your lab allow you to collect appropriate data yet identify moribund animals and remove them from the study to minimize suffering?

We are currently continuing to develop methods for analysis of proteomic and glycomic samples at UGA's Complex Carbohydrate Research Center (CCRC) from our 2014 experimental work conducted at the Savannah River Ecology Lab's Low dose facility (LoDIF). We are maintaining a stock of Japanese Medaka fish at the Savannah River Ecology Lab's Par Pond Aquatic Laboratory for continued research under this AUP (A2013 05-018-Y2-AO).

PI Approval

Signature of Principal Investigator: <-- Electronically Signed by Olin Rhodes -->

Date: 2015-03-20

The Attending Veterinarian verifies that the elements of this proposal have been assessed regarding the use of appropriate techniques in utilization of animals and that consultation with the PI will occur as necessary to resolve issue to minimize pain and distress.

Signature of Attending Veterinarian: <-- Electronically Signed by Leanne Alworth -->

Date: 2015-03-22

Final Approval

Received Final Approval on: 2015-04-01

Revision Comments:

No Revision Comments made for this Renewal.

Amendments

A2013 05-018-A1

1) Requested Modification

Provide details of the amendment to the approved AUP listed above. If more than one modification is requested, please number them.
Note: To request additional animals, please see section 2 of this form. For personnel modifications, please see section 3.

I am adding new personnel and removing one individual who has left employment at UGA.

2) Additional Animal Information

Species::(US Government Principles, Principle #3 Guide, p 12 AW Regs 9 CFR Part 2, sec 2.31 Internal record keeping/reporting data)	Choose a Species... ▼
Strain:	No Strains Available ▼
Highest Use Category:	Category A ▼
Sex:	Male ▼
Quantity (Numerical Only):	▼
Housing Location:	Choose a Facility...
Weight Range:	
Age Range:	
Preferred Vendor:	Choose a Vendor... ▼
Is the use of this species covered by the USDA Animal Welfare Act?	Yes No

3) Additional Personnel

Project Roster: Please provide the names of all additional individuals who will work with animals on this project to the IACUC. You do not need to include the staff of the facility in which your animals will be housed.

Occupation Health Program: Supervisors must enroll their employees in the OVPR Occupational Health and Safety Program. Please enroll personnel by having them complete a "Risk Assessment/Animal Contact Health Surveillance Questionnaire", available at the OACU OHS Page.

Training: Supervisors are responsible for insuring that their employees are adequately trained both in the specifics of their job and in the requirements of the Federal Animal Welfare Act.

All individuals working with live vertebrate animals, including the protocol Principal Investigator (PI), must complete federally required training on the pertinent laws and regulations covered in the "IACUC 101" course and health and safety covered in "Staying Healthy While Working with Animals."

The PI is responsible for keeping this roster for these individuals current. If staff is added or removed from this project, please modify the protocol to reflect this change; this is an administrative change and does not requires full IACUC review.

NEW INFORMATION:

Please be advised, for new personnel you must click the plus sign (+) by each individual's name to open a textbox in which you must provide the roles, responsibilities, and relevant training.

PERSONNEL ROSTER:
Olin Rhodes *(PI)* [-]

Describe this persons role, responsibilities and any relevant training.

Dr. Rhodes is the project PI and Director of the UGA Savannah River Ecology Laboratory. Dr. Rhodes is a Full Professor in the Odum School of Ecology and serves as the direct supervisor to Dr. Shem D Unger, who is a postdoctoral researcher at SREL. Dr. Rhodes is a population genetecist with 30 years of experience working with wildlife. He will be responsible for experimental design, overseeing research, supervising as a PI.

Training Courses Completed:

Course Name	Completion Date	CEUs
Continuing Education for Animal Research Credit	2019-03-11	1.00
UGA IACUC 101	2018-11-28	1.00
Sharps Training	2018-09-19	0.50
OHSP Decline to Participate	2016-06-08	0.00
Research Occupational Health Enrollment	2016-06-08	0.00
Continuing Education for Animal Research Credit	2016-03-01	1.00
UGA IACUC 101	2016-01-21	1.00
UGA IACUC 101 Refresher	2016-01-21	1.00
UGA IACUC 101	2013-02-13	1.00
Staying Healthy while Working with Laboratory Animals	2013-02-13	1.00

REBECA JUAREZ *(Added to AUP)* [-]

Can edit this submission form and draft amendments/renewals for this protocol: ○ Yes ◉ No

Describe this persons role, responsibilities and any relevant training.

Becky Juarez is a Research Technician in Dr. Rhodes laboratory with extensive experience (~1 year full time) in Medaka Culture. She will be responsible for day to day fish culture and maintenance of all fish habitats. Becky has a MS degree in wildlife ecology and management.

Training Courses Completed:

Course Name	Completion Date	CEUs
Occ Health Update	2016-11-30	0.00
Occupational Health and Safety Enrollment	2016-05-02	0.00
Staying Healthy while Working with Laboratory Animals	2016-04-21	1.00
UGA IACUC 101	2016-04-20	1.00

Xiaoyu Xu *(Added to AUP)* [-]

Can edit this submission form and draft amendments/renewals for this protocol: ◉ Yes ○ No

Describe this persons role, responsibilities and any relevant training.

Dr. Xu will be responsible for designing and assisting with exposure experiments for medaka to mercury. Dr. Xu has extensive experience with analysis of mercury exposure in aquatic organisms and has designed and undertaken experiments involving aqueous exposure of medaka to mercury as a postdoc.

Training Courses Completed:

Course Name	Completion Date	CEUs
Research Occupational Health Enrollment	2019-04-18	0.00
UGA IACUC 101	2019-04-17	1.00
Sharps Training	2019-02-25	0.50
Staying Healthy while Working with Laboratory Animals	2019-02-25	1.00
Occupational Health and Safety Enrollment	2016-04-28	0.00
Staying Healthy while Working with Laboratory Animals	2016-04-17	1.00
UGA IACUC 101	2016-04-17	1.00

REMOVED FROM AUP:
Shem Unger

PI Approval

Signature of Principal Investigator: <-- Electronically Signed by Olin Rhodes -->

Date: 2016-04-22

The Attending Veterinarian verifies that the elements of this proposal have been assessed regarding the use of appropriate techniques in utilization of animals and that consultation with the PI will occur as necessary to resolve issue to minimize pain and distress.

Signature of Attending Veterinarian: <-- Electronically Signed by Leanne Alworth -->

Date: 2016-04-26

IACUC Member Approval

Signature of IACUC Member: <-- Not Signed by IACUC Member -->

IACUC Chair Approval

Signature of IACUC Chair: <-- Not Signed by IACUC Chair -->

Final Approval

Received Final Approval on: 2016-05-06
